# Drug Design Strategies for the Treatment of Viral Disease. Plant Phenolic Compounds and Their Derivatives

**DOI:** 10.3389/fphar.2021.709104

**Published:** 2021-07-30

**Authors:** Monika Kowalczyk, Aleksandra Golonko, Renata Świsłocka, Monika Kalinowska, Monika Parcheta, Artur Swiergiel, Włodzimierz Lewandowski

**Affiliations:** ^1^ Laboratory of Biotechnology and Molecular Engineering, Department of Microbiology, Institute of Agricultural and Food Biotechnology—State Research Institute, Warsaw, Poland; ^2^ Department of Microbiology, Institute of Agricultural and Food Biotechnology—State Research Institute, Warsaw, Poland; ^3^ Department of Chemistry, Biology and Biotechnology, Bialystok University of Technology, Bialystok, Poland; ^4^ Faculty of Biology, University of Gdansk, Gdansk, Poland; ^5^ Institute of Agricultural and Food Biotechnology—State Research Institute, Warsaw, Poland

**Keywords:** antiviral activity, antiviral drugs, phenolic compounds, metal complexes, mechanism of action

## Abstract

The coronavirus pandemic (SARS CoV-2) that has existed for over a year, constantly forces scientists to search for drugs against this virus. *In silico* research and selected experimental data have shown that compounds of natural origin such as phenolic acids and flavonoids have promising antiviral potential. Phenolic compounds inhibit multiplication of viruses at various stages of the viral life cycle, e.g., attachment (disturbance of the interaction between cellular and viral receptors), penetration (inhibition of viral pseudo-particle fusion to the host membrane), replication (inhibition of integrase and 3C-like protease), assembly and maturation (inhibition of microsomal triglyceride transfer protein (MTP) activity hydrolysis) and release (inhibition of secretion of apolipoprotein B (apoB) from infected cells). Phenolic compounds also indirectly influence on the viral life cycle by affecting the host cell’s biochemical processes that viruses use for their own benefit. Phenolic compounds may inhibit the proteasomes and cellular deubiquitinating activity that causes an increase in the ubiquitinated proteins level in host cells. This, in turn, contributes to the lowering the available ubiquitin molecules that viruses could use for their own replication. One of the drug design strategy for the treatment of viral diseases may be an enhancement of the antiviral properties of phenolic compounds by metal complexation. Many studies have shown that the presence of a metal ion in the structure can significantly affect the affinity of the compound to key structural elements of the SARS CoV-2, such as M^pro^ protease, RNA-dependent RNA polymerase (RdRp) and spike protein. We believe that in the era of coronavirus pandemic, it is necessary to reconsider the search for therapeutics among well-known compounds of plant origin and their metal complexes.

## Introduction

The coronavirus pandemic (SARS CoV-2, severe acute respiratory syndrome coronavirus 2) that has spread worldwide since March 2020, has become and still continues to be the cause of the deaths of many millions of lives. For this reason, many countries took drastic measures to limit the spread of the virus (Jankun, 2020). Despite the development of vaccines it is imperative to search for cost-effective drugs that will prevent the disease and treat it effectively (Orfali et al., 2021).

Phenolic compounds are the most common group of substances in the plant kingdom (Rodriguez et al., 2015). They are derived from the secondary plant metabolism (Chávez et al., 2006). Phenolic compounds are present in vegetables, fruits, most legumes, many food grains, seeds, leaves, flowers, stems, roots, barks, wine (red wine), tea, honey, chocolate, and forages (Kamboj et al., 2012). They protect cells against ultraviolet radiation, oxidative stress and pathogen attack. Polyphenols include phenolic acids, flavonoids (flavonols, catechins (flavanols), flavones, isoflavones, flavanones, chalcones (flavanone isomers) and anthocyanins), which are the most numerous group of phenolic compounds, polyphenolic amides and other compounds such as lignans or stilbenes (Rodriguez et al., 2015; Annunziata et al., 2020). Many phenolic compounds present in natural products in the form of glycosides. More than 8,000 structures of phenolic compounds are known. These can be simple molecules such as phenolic acids or highly polymerized substances such as tannins (Rodriguez et al., 2015). Research showed that phenolic compounds exhibited a number of pharmacological effects, such as antioxidant, chelation of metal ion, vasodilatation, anti-inflammatory, antiallergenic, antitumor, antibacterial and antiviral activities (Chávez et al., 2006; Kamboj et al., 2012). Our studies on the phenolic compounds have shown that there is a significant dependence between the molecular structure of metal complexes of phenolic compounds and their antioxidant and antimicrobial properties. Complexation with metal ions with high ionic potential such us Ln (III), Y(III), Cr(III) and Fe(III) enhance the antioxidant properties of phenolic compounds even by up to ten times, due to the delocalization of the electronic charge distribution over the molecules (Świsłocka et al., 2019; Parcheta et al., 2021).

Studies revealed that natural products reach in phenolic compounds such as strawberries, apples, grapes as well as fruit juices possessed antiviral properties against herpes simplex virus (HSV), poliovirus, Coxsackie virus B5 and echovirus 7 (Ahmad et al., 2015). The polyphenols that have antiviral activity are among others caffeic acid, quercetin and myricetin (Mani et al., 2020). Because phenolic compounds demonstrated potent activity against a wide range of viral infections, there is a great interest in the evaluation of their activity against SARS-CoV-2. What is very important, the scientists reported that chemical modification of phytochemicals could increase their antiviral properties (Mani et al., 2020). The literature reports showed that metal ions or metal-containing compounds also exhibit antiviral activity (Ahmad et al., 2015).

We propose a new strategy for the design of antiviral drugs, also against the SARS CoV-2, consisting in metal complexation of phytochemicals, because both phenolic compounds and metal ions exhibit strong antiviral activity. We suppose that the metal complex may be even more effective than ligand and metal ion alone. We base our assumptions on the fact that some metals have the ability to increase the activity of conventional antiviral drugs (Varadinova et al., 2001; Valks et al., 2006; Shahabadi et al., 2012). Thus, it is likely that metals will enhance the antiviral activity of phenolic compounds. Moreover, complexation of antioxidants with selected metal ions increases their effectiveness (Świsłocka et al., 2019; Kalinowska et al., 2020; Kalinowska et al., 2021; Parcheta et al., 2021; Samsonowicz et al., 2021). In addition, metal complexation of phenolic compounds raises their lipophilicity and bioavailability (Samsonowicz et al., 2015; Kalinowska et al., 2018; Świderski et al., 2020), and also gives the opportunity to introduce beneficial micro- and macroelements into the compound (Świderski et al., 2020).

This review aims to describe the antiviral properties and mechanism of action against viral infections of various phenolic compounds and their derivatives, as well as their metal complexes, which are possibly also active against the SARS-CoV-2. In this review, we will explain why we believe that complex compounds of metals and phytochemicals may have even better antiviral properties than phenolic compounds alone. This review may be a very valuable source of information that can be put into practice in the future to design this type of drugs. If experimental research proves the high effectiveness of the compounds of metals and phytochemicals, it could be of great importance in combating the SARS-CoV-2 pandemic.

The present work is part of a wider topic (grant no. 2018/31/B/NZ7/03,083 financed by the National Science Centre), in which we study the relationship between the molecular structure and biological activity of selected ligands and their complexes of significant importance in medicine and pharmacy.

## Why is Metal Complexation of Phenolic Compounds Likely to Increase Their Antiviral Properties?

### Some Metals Have Antiviral Properties

It has been known for many years that some metals, such as Zn(II), Cu(II), Mg and Mn(II) have antiviral properties (Perrin and Stünzi, 1981; Chaturvedi and Shrivastava, 2005; Krenn et al., 2009; Ishida, 2018a; Ishida, 2018; Ishida, 2019). Metal ions play an important role in the survival and pathogenesis of a large group of viruses. Metal ions participate, among others in the maturation of viral genomic RNA, reverse transcription, protection of newly synthesized DNA and integration of viral DNA into the host cell DNA (Chaturvedi and Shrivastava, 2005). Zinc, magnesium and copper ions most often bind with viral proteins. Their excess can cause inhibition of virus production (Perrin and Stünzi, 1981; Chaturvedi and Shrivastava, 2005; Ishida, 2018). Attachment a metal ion to a protein via binding groups causes structural differentiation and changes in the electronic charge distribution of the protein, which in turn changes its reactivity. Similarly, protein functional groups attached to a metal ion change its reactivity. Analysis of the structure of metal binding to viral proteins will certainly be useful in the design of virus inhibitors (reverse transcriptase—RT, protease, integrase, nucleoside inhibitors) (Perrin and Stünzi, 1981; Chaturvedi and Shrivastava, 2005; Ishida, 2018). Studies have shown that Zn(II) ions ihibited the *in vitro* activity of nidovirus polymerases, the action of the SARS-CoV RNA-dependent RNA polymerase (RdRp) and synthesis of foot-and-mouth disease virus (FMDV) components such as RNA and procapsid (Krenn et al., 2009; Ishida, 2018). In addition, zinc ions are potent inhibitors of the fusion stage of several alphaviruses (Ishida, 2018a). Zn(II) and Cu(II) ions at a concentration of 0.5 mM inhibited the growth of FMDV in cell culture. These metal ions prevented the formation of the viral capsid protein because they interfered with the cleavage of large precursors of virus-specific protein (Perrin and Stünzi, 1981). Other physiologically relevant cations, such as Mn(II), inhibited the action of human immunodeficiency virus (HIV) reverse transcriptase (RT) (Ishida, 2018a). Studies have shown that each of the stages of the virus’s life cycle, i.e., attachment, penetration, replication, assembly and release, may become a potential target for new antiviral drugs (Ishida, 2019).

### Certain Metals Enhance the Effectiveness of Conventional Antiviral Drugs

The activity of the pyridine-2-carbaldehyde thiosemicarbazone (HFoTsc) (Figure 2A) and its six complexes containing platinum (II) or palladium (II) ions [PtCl(FoTsc)], [Pt(HFoTsc)_2_]Cl_2_, [Pt (FoTsc)_2_], [PdCl(FoTsc)], [Pd(HFoTsc)_2_]Cl_2_ and [Pd(FoTsc)_2_] against HSV-1 in infected cell cultures were studied. Most of the tested complex compounds, except [PdCl(FoTsc)], inhibited the growth of virus and showed structure-dependent activity. The researchers found that the antiviral properties of the complex depended on both the type of metal ion and the ligand (Varadinova et al., 2001).

It has been confirmed that zinc(II) increased the antiviral activity of the bicyclam derivatives. It turned out that EC_50_ values (effective concentration which caused a 50% reduction of the cytopathic effect induced by HIV-1 (IIIB strain) in MT-4 cells) for the drug–AMD3100 [the octa HCl salt of 1-1′-(1,4-phenylenebis-(methylene))-bis(1,4,8,11-tetraazacyclotetradecane)] and its complex with zinc (Zn_2_AMD3100) were 0.011 and 0.008 µM, respectively (Valks et al., 2006).

Integrase (IN) is a viral enzyme that catalyses the integration of viral DNA into the genome of the host cell. The antiviral properties (inhibition of integrase activity) of free ligands such as (2Z)-2-hydroxy-4-oxo-4-(3,5-benzyloxy)-phenyl-but-2-enoic acid and (Z)-3-hydroxy-1-phenyl-3-(1H-1,2,4-triazol-3-yl)prop-2-en-1-one, as well as their complexes with Mn(II) and Mg(II) were investigated by Bacchi et al. Bacchi et al. (2011). Both complexes and ligands blocked integrase activity very well.

Studies showed that the complex of the platinum (II) ion with the antiviral drug ribavirin [PtCl (DMSO) (N_4_N_7_-ribavirin)] H_2_O had a much stronger antiviral activity than ribavirin alone. It had greater affinity for DNA than the antiviral drug alone. Complexation of the metal ion with ribavirin caused ligand stiffening. The complex bound to DNA mainly through hydrophobic interactions, partly by intercalation (Shahabadi et al., 2012).

Rogolino et al. Rogolino et al. (2015) evaluated in *vitro* studies the inhibitory activity of free ligand–acylhydrazone and its complexes with Zn(II), Cu(II), Mn(II), Co(II), Ni(II) and Mg(II) against different DNA- and RNA-viruses (i.e., herpes simplex virus type 1 (HSV-1) strain KOS, HSV-2 strain G, vaccinia virus (VV) Lederle strain, human adenovirus type 2 (Ad-2), respiratory syncytial virus (RSV), vesicular stomatitis virus (VSV), Coxsackie B4 virus, Sindbis virus (SV), HIV-1 and HIV-2). The acylhydrazone and complex compounds tested (except for copper and cobalt complexes) were effective against DNA viruses such as HSV-1, HSV-2 and VV. The EC_50_ values obtained for acylhydrazone and complexes of acylhydrazone with Zn(II), Mn(II), Ni(II) and Mg(II) were in the micromolar range (Rogolino et al., 2015).

### Metal Complexation of Phenolic Compounds Could Increase Their Antioxidant Activity

Metal chelation of phenolic compounds has been widely described in the literature (Kalinowska et al., 2020). Metals can alter biological activity, including the antioxidant properties of ligands by affecting the molecular structure and charge density of phenolic compounds. Research showed that metal chelates can have higher antioxidant activity than phenolic compounds alone (Świderski et al., 2020). The highest antioxidant activity compared to free phenolic compounds was noted in the case of La(III), Mg(II) complexes of apigenin, Cu(II), Fe(II), La(III), Ce(IV) and oxovanadium (IV) complexes of chrysin, calcium complex of gentisic acid and oxovanadium complex of luteolin (Kalinowska et al., 2020). The research by Samsonowicz et al. Samsonowicz et al. (2021) proved that Cu(II) complex of ursolic acid was characterized by greater antiradical activity against DPPH^**•**^ than the ursolic acid alone. It was observed that the higher the concentration of the tested compounds, the greater their antioxidant activity was (Samsonowicz et al., 2021). Świsłocka et al. Świsłocka et al. (2019) claimed that alkali metal (Li, Na, K) rosmarinates exhibit better antioxidant activity than rosmarinic acid. Unfortunately, not all metal ions have the ability to increase the antioxidant properties of phenolic compounds. It has been reported that cadmium complex of quercetin showed lower antioxidant activity than free quercetin (Ravichandran et al., 2014). Fe(III) complex of luteolin and Fe(III) complex of baicalein exhibited less scavenging activity than free ligands (Samsonowicz et al., 2017). The antioxidant properties of phenolic compounds are determined, among others, by the position and number of hydroxyl groups in the aromatic ring, the degree of the electronic charge delocalization, the length of the conjugated double bond system, as well as the aromatic properties of the phenolic compounds, which are measured using the aromaticity indices and donor-acceptor properties described by the LUMO energy (Lowest Unoccupied Molecular Orbital) and HOMO energy (Highest Occupied Molecular Orbital) (Świderski et al., 2020). With increase of the HOMO orbital energy value, the better are electron donating activities (Lewandowski et al., 2020). The difference between the energy of HOMO-LUMO orbitals is known as the energy gap. It describes the affinity of molecule to participate in free radical scavenging process (Borges and Castle, 2015; Kowczyk-Sadowy et al., 2015). The smaller is the value of the energy gap, the higher is the capacity of molecule to take part in radical scavenging reaction, thus energy gap is the measure of the chemical reactivity of drugs and their cytotoxicity, which is involved in prediction of the active site placement in the drug molecule (Islam, 2015). Small energy gap provides high electron donating capacity of compound, which explains the high antioxidant activity of compounds. Gökalp compared the energy gap of phenolic compounds obtained from olive oil such as 1,4-hydroquinone, semiquinone, 1,4 benzoquinone calculated using density functional theory (DFT). 1,4-hydroquinone had the biggest energy gap and showed the greatest stability among cited compounds, while 1,4-benzoquinone had the greatest reactivity due to the small energy gap value (Gökalp, 2017). The studies over the inhibition of the 3C-like protease proved, that energy gap is the most important factor among lipophilicity and dipole moment in assessing the inhibitory capacity of the drug (Fong, 2020). One of the important descriptors describing the reactivity properties of compounds is dipole moment. According to Świsłocka et al., with the increase od dipole moment value the polarity of compounds grows (Świsłocka et al., 2019). The value of dipole moment also provides an information about the interaction with the active site of enzyme, as the dipole moment forms an index of reactivity (Sadasivam and Kumaresan, 2011). The activity of phenolic compounds can be estimated by calculation of the bond dissociation energy (BDE) of O-H bond. The lower is value the BDE, the higher is activity of studied compound, due to facilitation of O-H bond breakage. Metal complexation of ligands may contribute to the diminution of the BDE value in comparison with ligand alone, e.g., complexation of chlorogenic acid with zinc, enhanced the hydrogen abstraction of catechol moiety, to which zinc atom was introduced (Kalinowska et al., 2020). This mechanism may explain the antioxidant mechanism of action of metal complexes. Another descriptor of the activity of compounds is ionization potential (IP). There is a dependency between IP and HOMO orbital energy–IP value decrease with the increase of HOMO orbital energy value, which facilitate the tendency to donate the electrons and leads to enhance the reactivity of compound (Kalinowska et al., 2020). On the other hand according to Lewandowski et al., very small values of ionization potential may contribute to alteration antioxidant properties into prooxidant (Lewandowski et al., 2020). Probably the type of metal cation influences the antioxidant properties of phenolic compounds. Metal parameters that may determine the biological properties of the ligand can be ionic radius, electronegativity, ionic potential and effective charge of metal ion (Świderski et al., 2020). In case of alkali metals the perturbation of electronic system of molecules grows with the ionic radius, like in series Li → Na → K (Świsłocka et al., 2019).

### Complexation of Phenolic Compounds With Metals Favourably Changes Their Lipophilicity, Water-Solubility, Bioavailability, Stability and Also Allows the Introduction of Important Macro- and Microelements Into the Compound

Lipophilicity is a very important parameter of a compound that determines its ability to cross through the biological membranes (Kalinowska et al., 2020). Lipid-soluble substances can pass through the cell membrane. Tweedy’s chelation theory says that the coordination reduces the polarity of metal ions by partially sharing the metal ion with donor groups and possible delocalization of the π-electron charge over the chelate ring system (Kalinowska et al., 2014). The formed complex compounds have a more lipophilic character compared to ligand alone and therefore they can more easily penetrate the lipid layers of the cell membrane (Kalinowska et al., 2014). It has been proven that Li, Na, K, Rb and Cs salts of 5-O-caffeoylquinic acid (chlorogenic acid) are more lipophilic than chlorogenic acid (Kalinowska et al., 2018; Kalinowska et al., 2014). Samsonowicz et al. Samsonowicz et al. (2021) reported that metal complexation is a well-known way to increase the solubility of parent ligand. Literature data showed that transition metal complexes with phenolic acids and flavonoids are characterized by higher bioavailability compared to pure ligands (Świderski et al., 2020). Moreover, it has been indicated that complex compounds have greater *in vitro* and *in vivo* stability than parent flavone (Samsonowicz et al., 2017). It is worth noting that phenolic compounds by binding with metals can become a source of micro- and macroelements important for human health (Świderski et al., 2020).

The promising results of research on the biological activity of metal complexes and polyphenols led us to consider the influence of metal complexation on the antiviral activity of phenolic compounds. Perhaps metal complexation of phenolic compounds will prove to be a strategy for designing new, effective drugs against SARS-CoV-2.

## Antiviral Activity of Phenolic Compounds and Their Derivatives

The antiviral activity of phenolic compounds is known and very well described. The compounds discussed show activity against a wide range of viruses. Therefore, among the compounds of plant origin, one can also expect those that will be effective against the SARS-CoV-2. Recent studies indicate that the antiviral activity of these compounds is influenced by the number of hydroxyl groups, substituents and the relative position of functional groups. Compounds having a greater number of hydroxyl groups show a higher antiviral activity. Moreover, it appears that compounds with a methyl group have a stronger antiviral activity than compounds with a methoxy group (e.g., 2-methylphenol> 2-methoxyphenol). Interestingly, the position of the methoxy group at the two and six positions on the aromatic ring in the phenolic compound increases its activity compared to the compound with the methoxy groups at the three and four positions on the aromatic ring (eg., 2,6-dimethoxyphenol> 3,4-dimethoxyphenol). Optimizing the structure of the phenolic compound may be a new strategy for designing antiviral drugs (Li et al., 2017).

The antiviral properties of various phenolic compounds and their mechanism of action are presented below.

### Phenolic Acids

**FIGURE 1:**
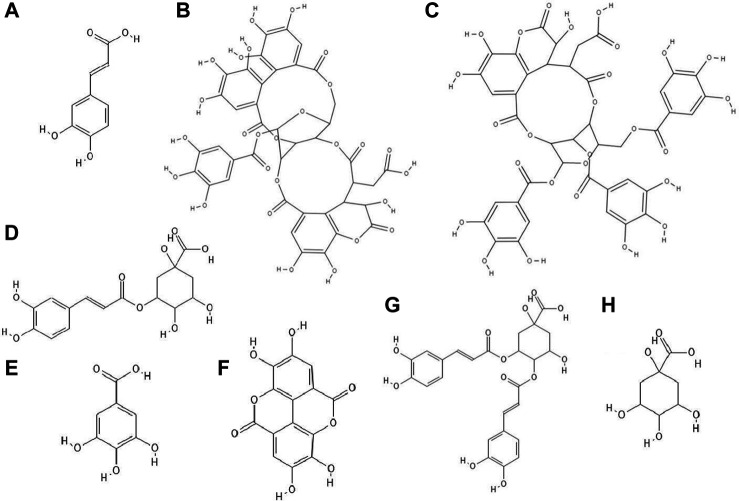
The structures of: caffeic acid **(A)**, chebulagic acid **(B)**, chebulinic acid **(C)**, chlorogenic acid **(D)**, gallic acid **(E)**, ellagic acid **(F)**, 3,4-dicaffeoylquinic acid **(G)**, quinic acid **(H)**.^1^ ^-^ ^8^

It has been revealed that caffeic acid (3,4-dihydroxycinnamic acid) (Figure 1A) at a concentration of 4 mM had the ability to inhibit the multiplication of HSV-1 (formation of progeny infectious virus) before the viral DNA replication was completed (viral DNA replication lasts between 3 and 6 h post infection). It is likely that this phenolic acid can specifically bond to the molecules necesarry for viral replication or directly to the virus. The researchers supposed that the free carboxyl group was responsible for the antiviral activity of caffeic acid (Ikeda et al., 2011).

The study discovered that chebulagic (Figure 1B) and chebulinic (Figure 1C) acids isolated from *Terminalia chebula* extract exhibited direct anti-HSV-2 activity *in vitro*. The IC_50_ (50% inhibitory concentration) values for these acids were 1.41 ± 0.51 μg/ml and 0.06 ± 0.002 μg/ml, respectively. Both compounds at concentrations up to 200 μg/ml caused only a 5% loss of viability of Vero cells. Chebulagic and chebulinic acids prevented the attachment and also penetration of virus into the cells. Probably these compounds have interacted with viral surface glycoproteins (Kesharwani et al., 2017).

Chlorogenic acid (CHA, 5-caffeoylquinic acid) (Figure 1D), a caffeic acid ester with quinic acid, due to its antiviral properties (anti-influenza virus, anti-parainfluenza virus and anti-respiratory syncytial virus) is used in China to treat viral infection of the upper respiratory tract. CC_50_ (50% cytotoxic concentration) of CHA against MDCK cell line was 364.30 ± 1.03 µM and this compound at concentrations of 10–100 µM reduced the cytopathic effect (CPE) in these cells caused by H_1_N_1_ and H_3_N_2_ viruses infections in a dose-dependent manner. In addition, chlorogenic acid showed the highest inhibition rates during the post-adsorption step of the H_1_N_1_ and H_3_N_2_ life cycle. Moreover, this acid prevented the release of viral components from infected cells by inhibiting of neuraminidase (NA), reducing the expression of viral NP protein (nucleoprotein) and its nuclear retention. Studies conducted on a lethal murine infection model have shown that CHA effectively increased survival rate of mice infected with both H_1_N_1_ and H_3_N_2_ (Ding et al., 2017).

Nutan and co-workers reported that gallic (Figure 1E) and ellagic acid (Figure 1F) derived from *Lagerstroemia speciosa* L. extract possessed anti-HIV activity. The tested compounds were antiviral at concentrations that were not cytotoxic to TZM-b1 and CEM-GFP cells. Gallic acid inhibited the activity of the enzyme reverse transcriptase, while ellagic acid inhibited HIV-1 protease. The studies showed that ellagic acid had no effect on the integrase activity (Nutan et al., 2013).

It has been observed that 3,4-O-dicaffeoylquinic acid (Figure 1G) derived from the extract *Laggera alata* after 8 days of incubation with HepG2.2.15 cells infected with hepatitis B virus (HBV) significantly inhibited the expression of HBsAg and HBeAg. HBsAg is the marker of HBV infection. On the other hand, HBeAg is an auxiliary marker informing about active HBV replication. Moreover, 3,4-O-dicaffeoylquinic acid at a concentration of 50 μg/ml reduced the amount of HBV cccDNA of HepG2.2.15 cells. In addition, this acid caused an upregulation of heme oxygenase 1 (HO-1), which leads to a reduction of the stability of the HBV core protein, thus preventing refilling of the nuclear HBV cccDNA (Wu et al., 2012).

### Flavonoids

**FIGURE 2:**
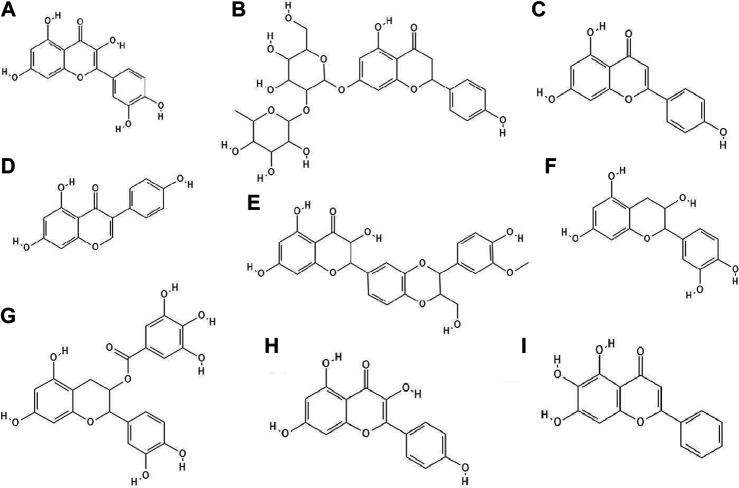
The structures of: quercetin **(A)**, naringin **(B)**, apigenin **(C)**, genistein **(D)**, silibinin **(E)**, epicatechin **(F)**, epicatechin gallate **(G)**, kaempferol **(H)**, baicalein **(I)**.^9^ ^-^ ^16^

It turned out that not only phenolic acids, but also flavonoids exhibited antiviral properties. The antiviral activity (against HSV-1 and parainfluenza virus type-3) of flavonoids such as quercetin (Figure 2A), naringin (Figure 2B), apigenin (Figure 2C), genistein (Figure 2D), silibinin (Figure 2E) and silymarin and phenolic acids such as caffeic, chlorogenic, gallic and quinic (Figure 1H) acids has been determined. All of the flavonoids screened showed activity against HSV-1. Naringin and apigenin were characterized by the strongest antiviral activity. They had the widest therapeutic range of 0.4–1.6 μg/ml. Genistein and phenolic acids such as gallic (0.05–0.8 μg/ml), chlorogenic (0.4–1.6 μg/ml) and quinic acid (0.4–1.6 μg/ml) had different activity against parainfluenza virus type-3. Most of the tested compounds possessed higher cytotoxicity to MDCK cells than acyclovir (1.6 μg/ml) (Özçelik et al., 2011).

**FIGURE 3:**
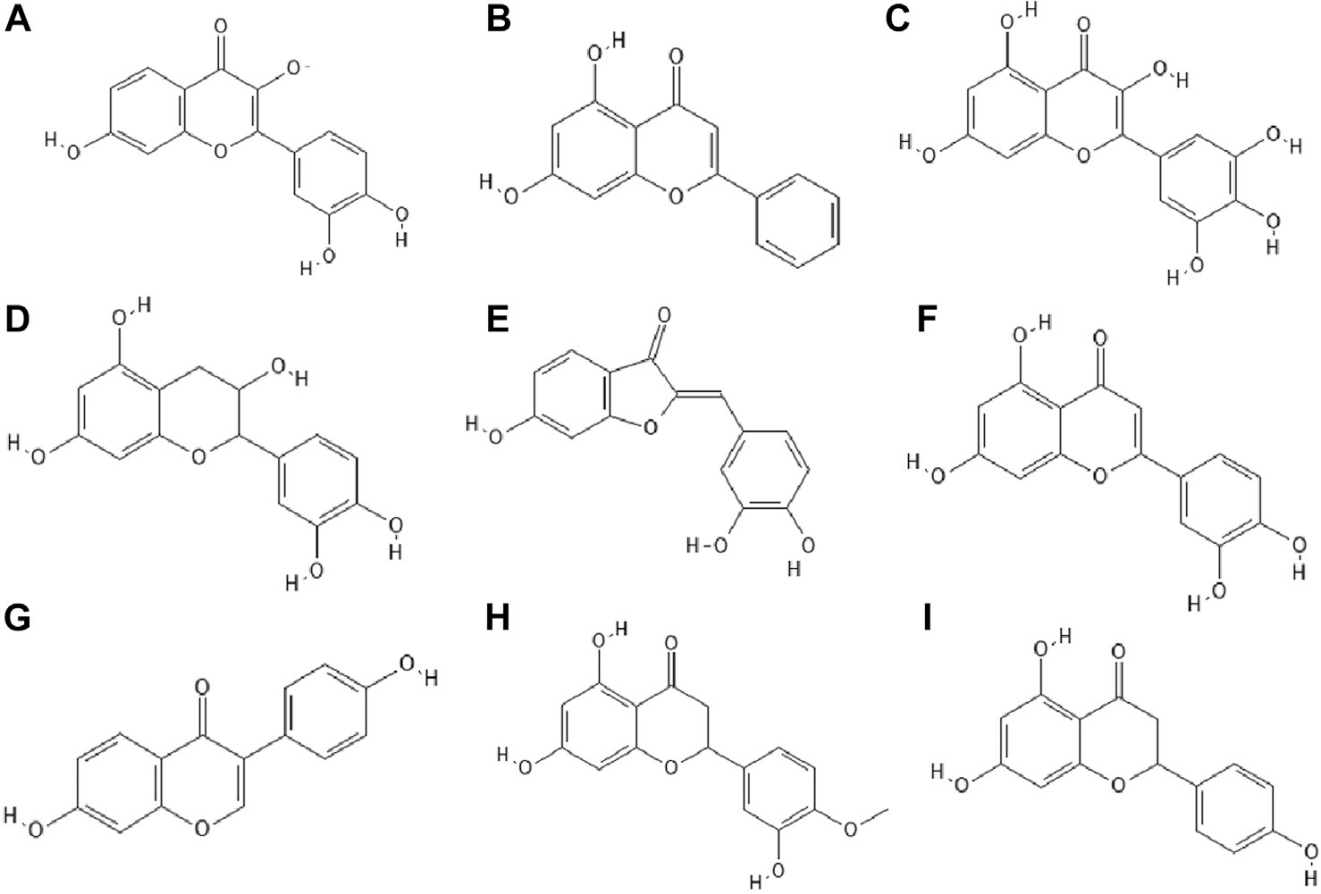
The structures of: fisetin **(A)**, chrysin **(B)**, myricetin **(C)**, catechin **(D)**, sulfuretin **(E)**, luteolin **(F)**, daidzein **(G)**, hesperetin **(H)**, naringenin **(I)**.^17^ ^-^ ^25^

14 out of 18 tested flavonoids belonging to five classes exhibited a potent anti-herpetic activity. The study showed that flavonoids such as epicatechin (Figure 2F), epicatechin gallate (Figure 2G) and kaempferol (Figure 2H) were characterized by strong antiviral activity, while quercetin, genistein, baicalein (Figure 2I), fisetin (Figure 3A), chrysin (Figure 3B), myricetin (Figure 3C) and catechin (Figure 3D) were moderately active against HSV-1 (results obtained by plaque reduction assay) (Lyu et al., 2005).

Interestingly, from the literature reports it appeared that the combination of certain phenolic compounds, e.g. those belonging to flavonols and flavones like luteolin and kaempferol, showed a synergistic effect against HSV (Ahmad et al., 2015). Moreover, some flavonoids, such as quercetin, increased the potency of the antiviral drug acyclovir (Ahmad et al., 2015).

It has been studied whether 25 different flavonoids had the ability to inhibit the neuraminidase (NA) activity of influenza virus. The highest antiviral activity was observed in aurones (e.g., sulfuretin (Figure 3E), followed by flavones (e.g., apigenin, luteolin (Figure 3F), flavon (ol)es, isoflavones (e.g., daidzein (Figure 3G), flavanone (ol)es and flavan (ol)es. The research showed that the 7-OH, 4'-OH, C4 = O, and C2 = C3 moieties were responsible for the inhibition of NA viral activity. On the other hand, the presence of the glycosylation group in the compound structure decreased the inhibition of neuraminidase activity (Liu et al., 2008).

In the study by Zandi et al. Zandi et al. (2011) quercetin showed significant activity against DENV-2 (Dengue virus type 2, New Guinea C strain–NGC strain) in Vero cells. This compound exhibited prophylactic activity and antiviral activity after post-adsorption stage. The authors supposed that the main mechanism of action of quercetin against DENV-2 was inhibition of intracellular replication of the virus (not virus attachment or entry to cells). Perhaps quercetin had the ability to inhibit RNA polymerase. The remaining bioflavonoids, such as daidzein and naringin, possessed weak anti-DENV-2 (daidzein exhibited antiviral activity after adsorption the virus to Vero cells; naringin blocked attachment and adsorption effects), or no antiviral activity at all like hesperetin (Figure 3H) (Zandi et al., 2011).

**FIGURE 4:**
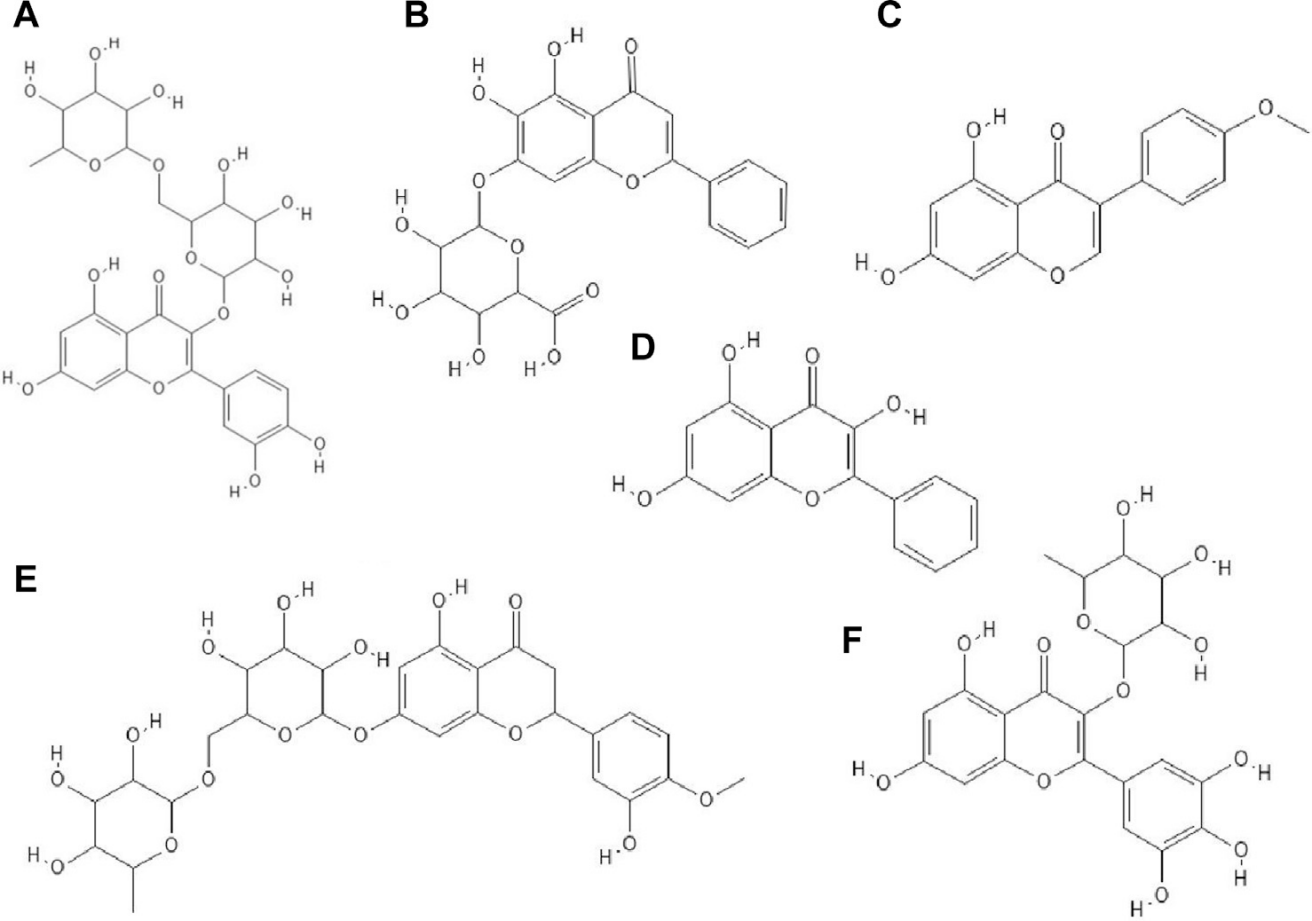
The structures of: rutin **(A)**, baicalin **(B)**, biochanin A **(C)**, galangin **(D)**, hesperidin **(E)**, myricetin 3-rhamnoside **(F)**.^26^ ^-^ ^31^

The antiviral properties (against DENV-2 (New Guinea C strain)) of fisetin, naringenin (Figure 3I) and rutin (Figure 4A) has been investigated. The research showed that fisetin possessed antiviral activity (inhibition of DENV-2 replication), but this flavonol did not exert direct virucidal effects on DENV-2, because no inhibition was observed when this compound was added directly to the virus suspension. Both naringenin and rutin did not inhibit viral replication in Vero cells, however naringenin acted directly toward the virus (Zandi et al., 2011a).

It has been checked whether ten flavonoid compounds (flavones: baicalein, baicalin (Figure 4B), apigenin, luteolin; isoflavones: daidzein, genistein, biochanin A (Figure 4C); flavonone: naringenin and flavonols: quercetin, galangin (Figure 4D) possessed activity against HCMV (human cytomegalovirus). Nine out of ten compounds tested had the ability to block virus replication. The flavonoids acted at concentrations lower than cytotoxic to human embryonic lung fibroblasts (HEL 299). The most potent antiviral compound was baicalein. This phenolic compound reduced viral DNA synthesis and the levels of HCMV early and late proteins. Pre-incubation of HCMV stocks with baicalein did not cause inhibition of virus replication, so this compound did not directly inactivate the virus particles. The authors also reported that hydroxylated flavonoids at position six in aromatic ring A had the highest anti-HCMV activity among the flavone and flavonol derivatives group (Evers et al., 2005).

Paredes et al. Paredes et al. (2003) reported that two flavanones, hesperetin and naringenin, had the ability to inhibit replication of neurovirulent strain of Sindbis virus (NSV), with naringenin having greater antiviral activity. This compound at a concentration of 25 μg/ml inhibited virus replication in 80% (Paredes et al., 2003).

### Derivatives of Phenolic Compounds

The glycoside group of hesperetin and naringenin, such as hesperidin (Figure 4E) and naringin did not possess antiviral properties. The authors noted that oxygenation on the 3’ positions at the B rings on the hesperetin skeleton caused a reduction in antiviral properties (Paredes et al., 2003).

Research has proved that kaempferol-3-O-β-D-xylopyranosyl (1 → 2) α-L-rhamnopyranoside and quercetin-3-O-β-D-xylopyranosyl (1 → 2) α-L-rhamnopyranoside exhibited anti-HSV-1 and anti-HSV-2 activities. The EC_50_ value for the kaempferol derivative was 7.4 μg/ml and 9.0 μg ml, respectively, and for the quercetin derivative 5.8 μg/ml and 36.2 μg/ml. The CC_50_ values for both compounds were higher than 200 μg/ml (Ürményi et al., 2016).

Myricetin derivatives such as myricetin 3-rhamnoside (Figure 4F) and myricetin 3-(6-rhamnosylgalactoside) have anti-HIV-1 activity *in vitro* studies. Both compounds inhibited RT activity (IC_50_ = 10.6 µM for myricetin 3-rhamnoside and IC_50_ = 13.8 µM for myricetin 3-(6-rhamnosylgalactoside)). Researchers claimed that glycosylated moiety can facilitate the entry of the phenolic compound into the cell, thereby increasing its antiviral activity (Ortega et al., 2017).

It has been noted that quercetin 3-O-[(6-O-sinapoyl)-β-D-glucopyranosyl-(1 → 2)-β-D-galactopyranoside] and quercetin 3-O-[(6-O-ferulolyl)-β-D-glucopyranosyl-(1 → 2)-β-D-galactopyranoside] inhibited HIV-1 integrase with IC_50_ values of 7 and 5 μM, respectively. Compounds bearing sinapoyl or ferulolyl group in the terminal glucose moiety were found to have higher antiviral activity than unsubstituted ones (Jiang et al., 2010).

Wagoner et al. Wagoner et al. (2010) determined which stages of the hepatitis C virus (HCV) life cycle are inhibited by silymarin. Their research proved that silymarin blocked the entry of viral pseudoparticles (HCVpp) into the cell and their fusion with liposomes. However, silymarin had no effect on HCVpp binding to cells. In addition, silymarin inhibited RNA production, genotype 2a NS5B RNA-dependent RNA polymerase (RdRp) activity, microsomal triglyceride transfer protein (MTP) activity, secretion of apolipoprotein B (apoB) from HCV-infected cells, virion production and cell-to-cell spread of virus (Wagoner et al., 2010).

### Phenolic Compounds in Extracts

**FIGURE 5:**
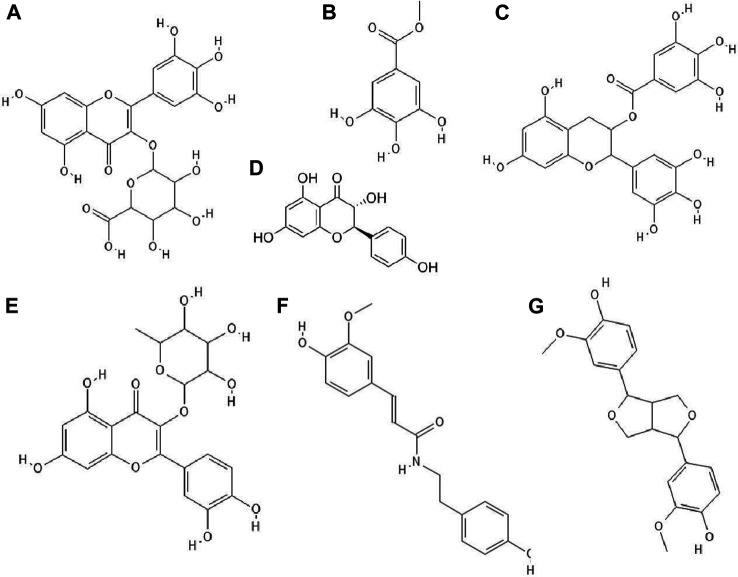
The structures of: myricetin-3-O-glucuronide **(A)**, methyl gallate **(B)**, epigallocatechin gallate **(C)**, dihydrokaempferol **(D)**, quercitrin **(E)**, N-trans-ferulolyl tyramine **(F)**, pinoresinol **(G)**.^32^ ^–^ ^38^ ^,^ ^44^ (the chemical structures of A, B, C, E, F, G are available at https://pubchem.ncbi.nlm.nih.gov/, National Center for Biotechnology Information, 2021, and the structure of D is available at Sigma-Aldrich https://www.sigmaaldrich.com).

Phenolic compounds isolated from methanol (70%) extract of *Diospyros lotus* fruits had anti-HIV-1 (IIIB strain) properties. The strongest antiviral activity was shown by gallic acid, followed by ellagic acid, myricetin-3-O-α-rhamnoside, myricetin-3-O-β-glucuronide (Figure 5A), methyl gallate (Figure 5B), myricetin and quercetin. Among the mentioned compounds, the most cytotoxic was ellagic acid (CC_50_ = 35.84 μg/ml) (Rashed et al., 2012).

Anti-RSV (respiratory syncytial virus), anti-CVB3 (Coxsackie virus B3) and anti-HSV-1 of phenolic compounds isolated from ethanol extract of the whole plants of *Origanum vulgare* were tested. It has been disclosed that most of the compounds showed no activity against the mentioned viruses. For example, acacetin 7-O- [4'' -O-acetyl-β-D-apiofuransyl-(1 → 3)]-β-D-xylopyranoside possessed weak activity against RSV A2 strain (IC_50_ = 81.7 µM), while acacetin 7-O- [6 '''-O-acetyl-β-D-galactopyranosyl-(1 → 2)]-β-D-glucopyranoside showed weak activity against HSV-1 (IC_50_ = 38.5 µM) (Zhang et al., 2014).

The hydro-ethanolic extract of *Limonium densiflorum* rich in phenolic compounds possessed activity against HSV-1. The antiviral activity of compounds isolated from this extract were gallic acid, epigallocatechin gallate (Figure 5C), dihydrokaempferol (Figure 5D), quercitrin (Figure 5E), *N*-*trans*-ferulolyl tyramine (Figure 5F), pinoresinol (Figure 5G) and (myricetin 3-O-α-rhamnopyranoside and myricetin 3-O-L-arabinofuranoside. Gallic acid and epigallocatechin gallate exhibited strong anti-HSV-1 activity, whereas *N*-*trans*-ferulolyl tyramine and pinoresinol moderate properties. The remaining tested compounds did not possess antiviral activity. Scientists emphasized that phenolic compounds show high affinity for proteins, forming unstable complexes with them. Probably, these compounds interact with viral envelope glycoproteins, inhibit viral polymerase, which affects the synthesis of the viral genome (Medini et al., 2016).

**FIGURE 6:**
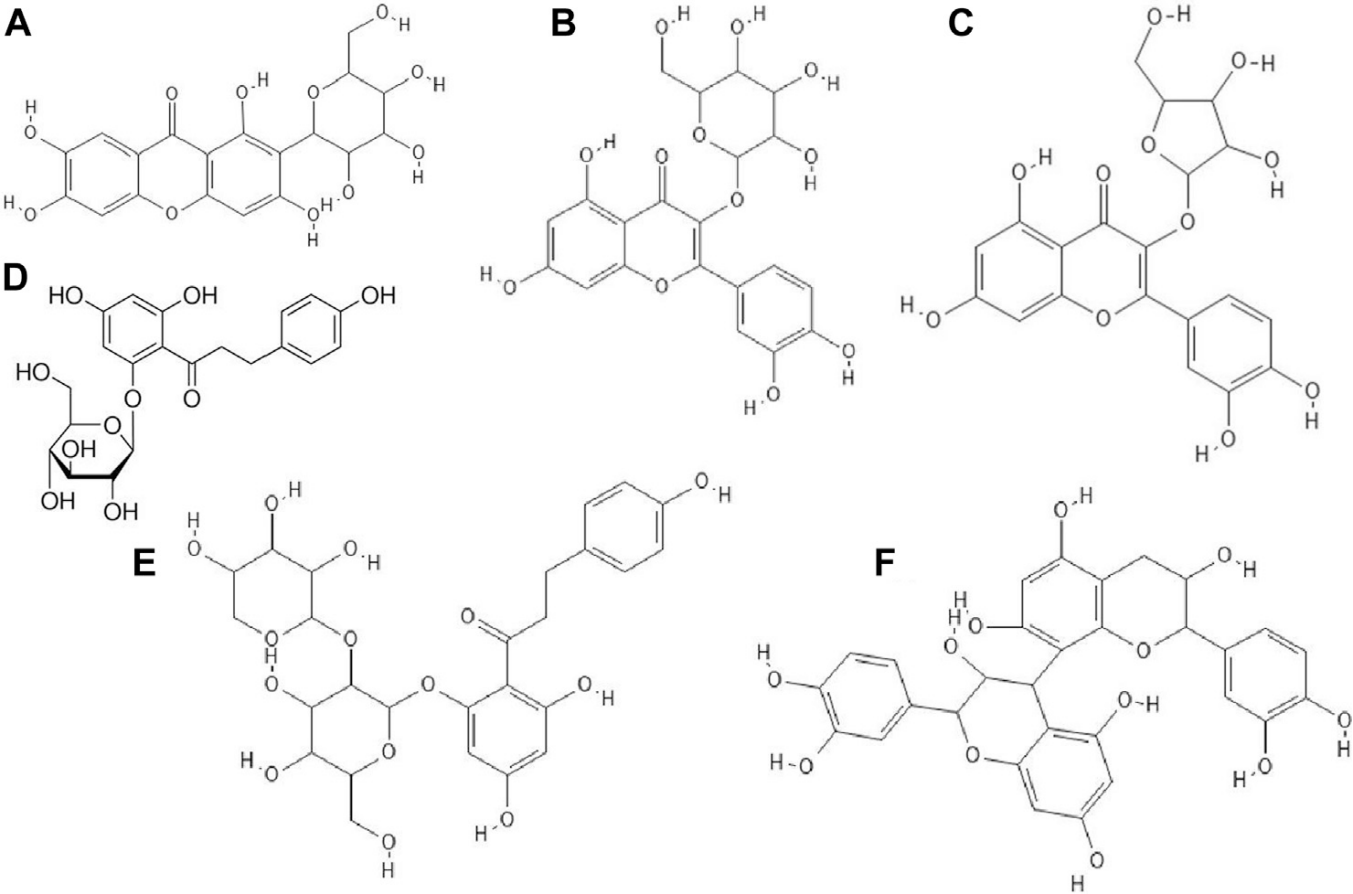
The structures of: mangiferin (1,3,6,7-Tetrahydroxyxanthone C2-β-D-glucoside, **(A)**, hyperin (quercetin 3-D-galactoside) **(B)**, avicularin (quercetin 3-O-α-L-arabinofuranoside) **(C)**, phloridzin (phloretin 2′-β-D-glucopyranoside), **(D)**, phloretin, **(E),** procyanidin B2 **(F)**.^39^ ^-^ ^43^ ^,^ ^45^

Certain phenolic compounds isolated from the ethanol extract of *Bombax malabaricum* flower such as kaempferol-3-O-(6”-O-E-p-coumaroyl)-β-D-glucopyranoside and mangiferin (Figure 6A) exhibited antiviral activity against RSV Long strain. Furthermore, kaempferol-3-O-(6”-O-E-p-coumaroyl)-β-D-glucopyranoside showed similar anti-RSV activity to ribavirin. The authors suggested that (di)hydrocinnamoyl functional groups were responsible for the strong antiviral activity (Zhang et al., 2015).

Suárez et al. Suárez et al. (2010) confirmed that methanolic and acetonic extracts of apple pomace showed antiviral properties against HSV-1 and HSV-2. These extracts were abundant in quercetin glycosides such as hyperin (Figure 6B) and avicularin (Figure 6C) followed by dihydrochalcones such as phloridzin (Figure 6D) and phloretin-2'-xyloglucoside (Figure 6E), phenolic acids, epicatechin and procyanidin B2 (Figure 6F). Methanolic and acetonic extracts of apple pomace inhibited virus replication at concentrations 9-fold lower than the cytotoxic concentrations. The authors speculated that the flavonoids were responsible for the antiviral properties of the tested extracts (Suárez et al., 2010).

**FIGURE 7:**
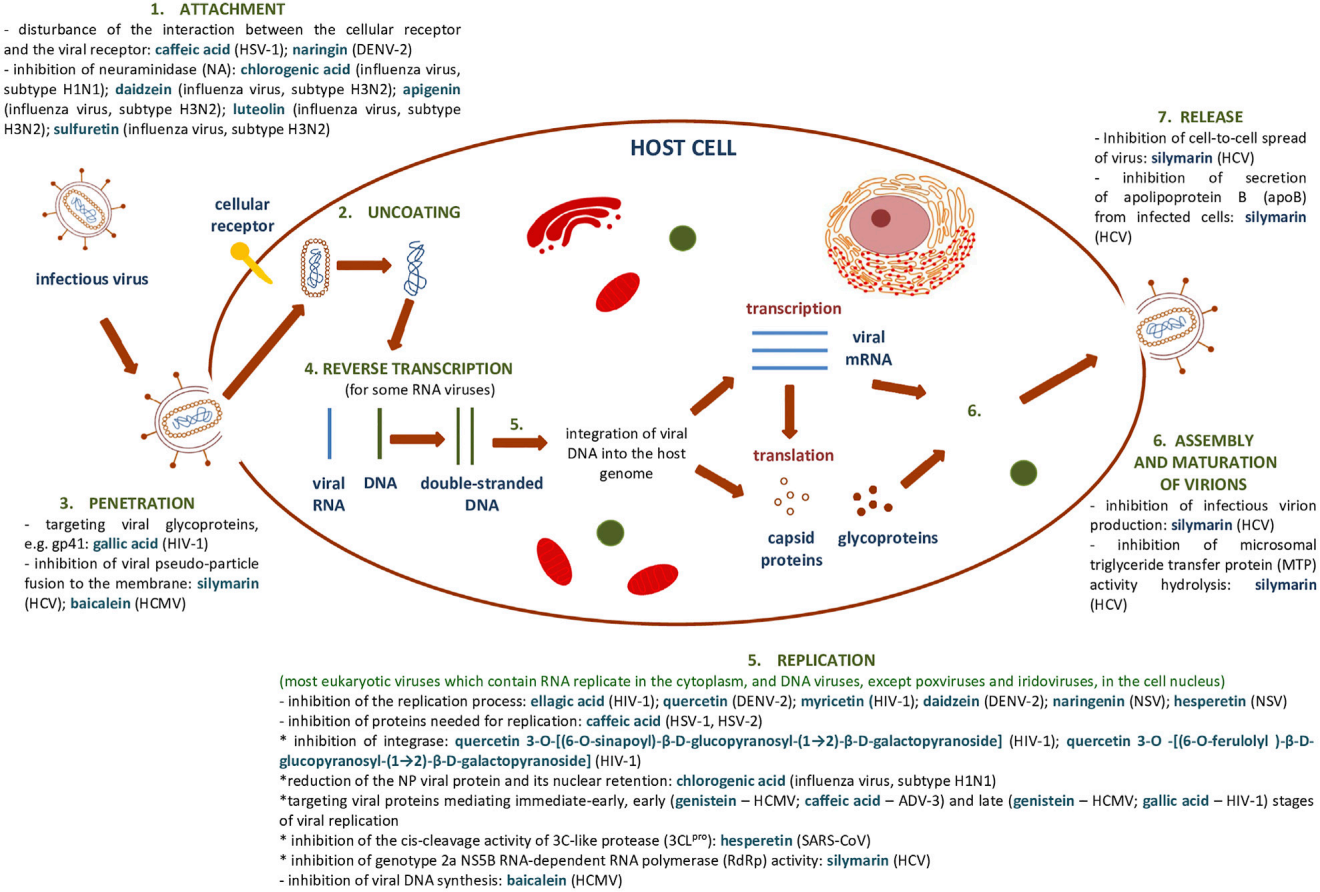
General scheme of the action of phenolic compounds on each stage of the viral replication cycle based on (Dustin et al., 2016) and collected literature in this review.

Supplementary Table S1, S2 summarize the results showing the antiviral activity of the phenolic compounds and their derivatives described above. Figure 7 shows a general scheme of the action of phenolic compounds on the virus based on the collected literature.

## Anti-Severe Acute Respiratory Syndrome Coronavirus 2 Potential of Natural Polyphenols Compounds

In the context of drug discovery for SARS-CoV-2, the first studies were largely based on *in silico* methods. This allowed for a preliminary evaluation of the use of potential structures against viral infection.

The rapid increase in the need to find effective drugs against COVID-19 (Coronavirus disease 2019) has contributed to greater interest in *silico* molecular modeling in screening, which allows the comparison of the affinity energies of thousands of molecules with viral targets (Zhang et al., 2020).

**FIGURE 8:**
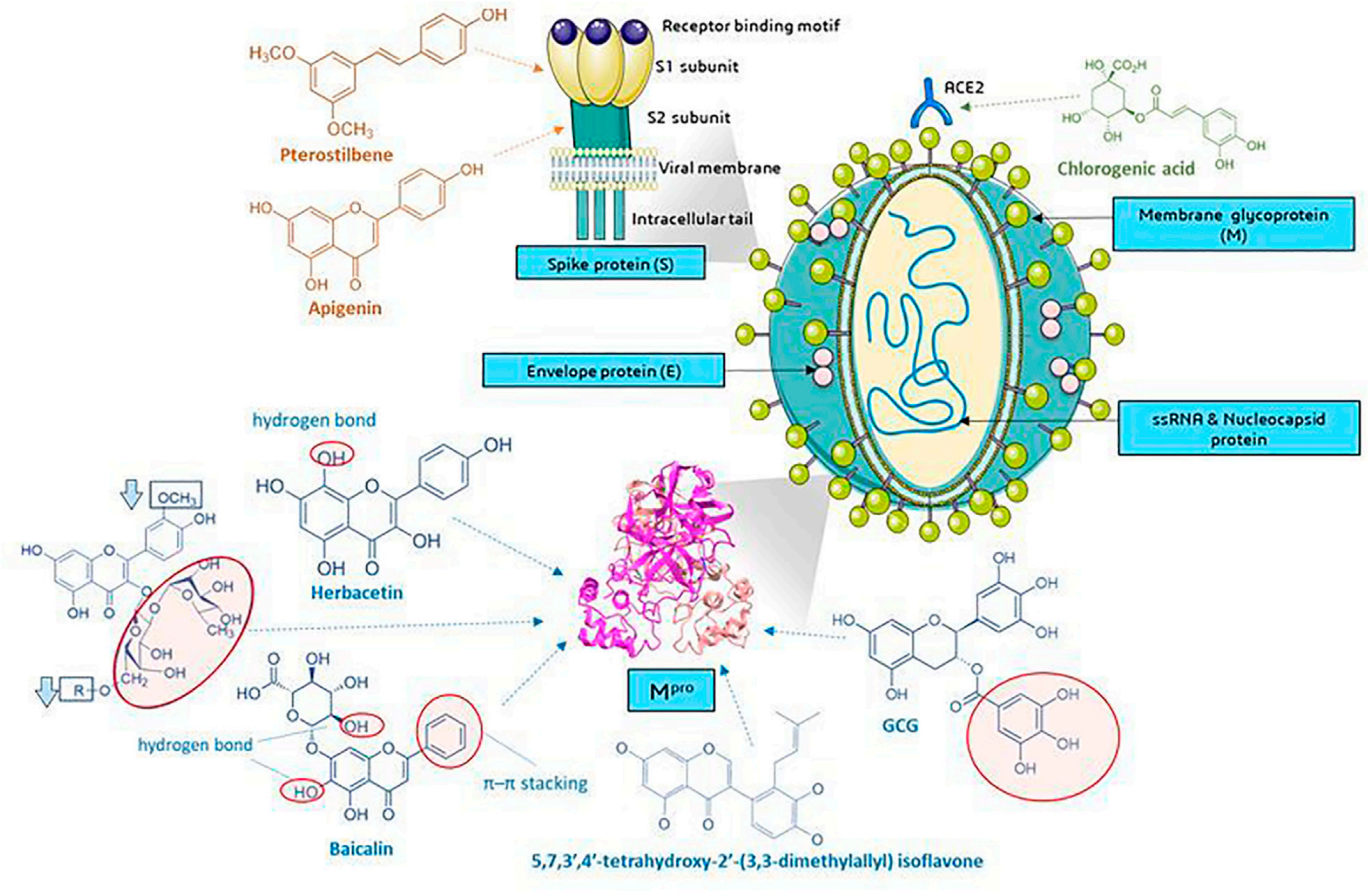
Molecular targets of selected phenolic compounds action on SARS-CoV-2 virus, based on described *in vitro* and *in silico* experiments. Spike protein. Molecular docking analysis and the putative binding sites of selected inhibitors on SARS-CoV-2S (spike protein) in C-terminal of S1 domain: Pterostilbene have lowest value of binding affinity -6.7 kcal/mol vs hydroxychlo-roquine -5.6 kcal/mol); S2 domain-apigenin -7.7 kcal/mol (Pandey et al., 2020). M^pro^ (figure presented PDB ID: 6LU7; The crystal structure of COVID-19 main protease in complex with an inhibitor N3). GCG has essential galloyl moiety, which forms four hy-drogen bond interactions with aminoacid residues (Nguyen et al., 2012), the 8-OH moiety present in the herbacetin molecule (Jo et al., 2019a). Baicalein–forms hydrogen bonds and π–π stacking between aminoacid residue and the phenyl moiety (Jo et al., 2020), flavonoids that increase (red circle) or decrease (rectangle) binding stability into N3 binding site in the COVID-19 main protease (Owis et al., 2020), similar as deccribed Cherrak et al., and da Silva et al., that glycosylated flavonoids display a stronger inhibitory activity than aglycones (Cherrak et al., 2020; da Silva et al., 2020; Rehman et al., 2020). *In silico* analyzes show the formation of stable bonds of chlorogenic acid with Gln325 and Gln42/Asp38 in ACE2 (Yu et al., 2020; Servier Medical Art, 2021). Graphics from https://smart.servier.com/.

Several current analyses have been based on the assumption of homology of SARS-CoV-2 and SARS-CoV infections (79.5% sequence identity) and the study of the affinity of natural compounds for the most important structural elements of SARS-CoV-2 (Figure 8). These elements are among others spike protein (S)—essential for entry of the virus into the host cells, S1 domain—the main antigen on the surface of the virion with the receptor binding domain (RBD) interacting with ACE2, S2-mediating the fusion between the viral membrane and the cell membrane, E envelope protein—the smallest structural protein of CoV, active in the assembly process and egress, membrane protein (M)—maintains the shape of the envelope, promotes IFN- β induction via Toll-like receptor related mechanism (Wang and Liu, 2016), the nucleocapsid protein (N)—surrounding and protecting the viral genome, able to modulate the internal environment of the host cells to a more favourable virus survival and is a potential target in vaccine design (Figure 8), and RdRp—RNA-dependent RNA polymerase. The site of coronavirus binding to host cells is the membrane enzyme ACE2 (angiotensin-converting enzyme 2) found in enterocytes, lung epithelium, kidneys and blood vessels. After virus attachment clathrin-dependent endocytosis occurs (Zhang et al., 2020a). The viral fusion protein allows the virus to attach to the ACE2 receptor and fuse with the host cell. Therefore one of the strategy is to the search for small molecules against COVID-19 that disrupt the viral S-protein-ACE2 interface (Smith and Smith, 2020).

### Role of Phenolic Compounds as the Virus S Protein-Host Angiotensin-Converting Enzyme 2 Receptor Interaction Inhibitor

It was found that among the analyzed structures, there were several natural ligands against the surface of the interaction of the SARS-CoV-2 protein with the ACE2 receptor. The calculated Vina Score for selected compounds is -7.1 for eriodictyol, -7.4 for luteolin and -7.3 quercitin. Eriodictyol is as a compound that forms twice as many interactions with the ACE2 receptor than the S protein (Smith and Smith, 2020). Eriodictyol, luteolin and quercetin have the potential to reduce virus-host interactions, with ACE-2. Muchtaridi et al. (Muchtaridi et al., 2020) described the most important functional groups responsible for ACE2 inhibition. In the review there was emphasized the important role of the location of hydroxyl groups in the benzene ring at the meta position (resorcinol structure) in the A-ring of the flavan ring which may disrupt hydrogen bonds between amino acid residues between ACE2 and S protein, as observed for phillyrin and chlorogenic acid. These compounds can be used as potential inhibitors of COVID-19 for further research (Muchtaridi et al., 2020; Yu et al., 2020). The QSAR (quantitative structure–activity relationship) analyzes indicated that the presence of the B-ring, the carboxyl groups in this ring and the 3-OH, 3′-OH and 5′-OH formation were key to the ACE inhibitory activity. The absence of these structures significantly reduced the ACE inhibitory properties of the compound (Guerrero et al., 2012). In review by Muchtaridi et al. Muchtaridi et al. (2020), the authors concluded that there was a correlation between ACE2 inhibition and the anti-inflammatory and antioxidant effects of phenolic compounds due to their structure (Muchtaridi et al. 2020). The presence of hydroxyl groups favors interactions with key residues, while the position and number of hydrogen bonds are important in the analysis of the inhibitory potential (Vijayakumar et al., 2020). Molecular docking studies performed by Ngwa et al. Ngwa et al. (2020) indicated that flavonoid molecules bind effectively to the ACE2 receptor, also compared to chloroquine, which has been studied in multiple clinical trials as an anti-COVID-19 drug. The lowest binding energy value was found for linebacker, which is patented universal therapeutic medication (−9.2 kcal/mol), and the remaining values were hesperetin (−9.1 kcal/mol)<myricetin (−8.9 kcal/mol)<caflanone (isocannflavin B) (−7.9 kcal/mol)<chloroquine in possible binding poses (−4.1/-4.7 kcal/mol) (Ngwa et al., 2020). Interestingly, *in vitro* studies indicated the inhibitory potential against virus entry factors, including ABL-2, AXL-2, cathepsin, pro-inflammatory cytokines IL-1β, IL-6, IL-8, Mip-1α, TNF-α. The authors of these studies also designed smart nanodrone, in which flavonoid acted as targeting moieties with the possibility of space-time distribution and treatment monitoring, which may ultimately be a method of direct delivery to the lungs (Ngwa et al., 2020).

### Phenolic Compounds as the Spike Protein Site-Blocking Factors

Another purpose, important from drug discovery side is spike protein. A docking analysis by Pandey et al. Pandey et al. (2020) suggested that some flavonoids bind to the C-terminus of the S1 domain (kaempferol: 7.4, curcumin -7.1, pterostilbene -6.2 kcal/mol) and the S2 domain of this protein (fisetin: 8.5, quercetin -8.5, isorhamnetin -8.4, genistein -8.2, luteolin -8.2, resveratrol -7.9, apigenin -7.7 kca/mol). In comparison, hydroxychloroquine, an FDA approved drug, binds to the S1 domain with an energy of -5.6 kcal/mol (Pandey et al., 2020). Also, Vijayakumar et al. Vijayakumar et al. (2020) noted that quercetin forms favorable bonds with spike protein with a −7.8 kcal/mol binding affinity with amino acid residues (Vijayakumar et al., 2020).

### Phenolic Compounds With the Inhibiting Activity Towards Viral Proteases in Host Cells

3CL^pro^ protease (M^pro^) has a high homology to the SARS-, SARS-2- and MERS-CoV proteases. The literature reported that the proteolytic activity of 3CL^pro^ SARS-CoV can be inhibited by apigenin, luteolin, quercetin, amentoflavone, quercetin, daidzein, puerarin, epigallocatechin, epigallocatechin gallate, gallocatechin gallate and kaempferol (Ryu et al., 2010; Nguyen et al., 2012; Schwarz et al., 2014; Jo et al., 2019a). There were significant differences in the IC_50_ values compared to the 3CL^pro^ of individual polyphenols: for apigenin this value was 280.8 ± 21.4, and for luteolin and quercetin it was 20.0 ± 2.2 and 23.8 ± 1.9 μM, respectively. These values were compared with other compounds extracted from *Torreya nucifera* leaf extract, with the lowest IC_50_ among biflavonoids for amentoflavone with IC_50_ 8.3 ± 1.2 and Ki = 13.8 ± 1.5 (noncompetitive) and ferruginol among diterpenoids (IC_50_ = 49.6 ± 1.5). Jo et al. Jo et al. (2019a) noted that the assessment of the inhibitory potential of phenolic compounds should be verified by the presence of Triton X-100, which prevents the nonspecific binding of the studied ligands to the aggregate and complex-forming proteases. According to Ryu et al. Ryu et al. (2010), the strongest, flavonoid-amentoflavone, turned out to be ineffective in inhibiting the proteolytic activity of SARS-CoV 3CL^pro^ in the presence of Triton-X 100. In the evaluation of the interaction of flavonoids with the catalytic site of the enzyme, Jo et al. Jo et al. (2019a) indicated that the presence of the 8-OH moiety present in the herbacetin molecule (3,4', 5,7,8-pentahydroxyflavone) (IC_50_ = 33.17 µM) was essential for high binding affinity in the S1 and S2 sites. Among the glycosylated flavonoids, the high affinity of rhoifolin (IC_50_ = 27.45 µM) and pectolinarin (IC_50_ = 37.78 µM) was reported (Jo et al., 2019a). Th molecular docking showed that they have a wide range of binding affinity to CL^pro^ due to their hydrophobic aromatic rings and hydrophilic hydroxyl groups and presence of carbohydrate groups influences severely to the binding affinity and mode of the chromen-4-one moiety. Earlier studies by this author on the inhibitory activity of MERS 3CL^pro^ indicate that flavonol and chalcone are the key structures of the flavonoid in binding to the catalytic site of the enzyme (MERS-CoV 3CL^pro^). In addition, docking comparisons indicated that hydroxymethyl group of the glucoside moiety in quercetin 3-β-d-glucoside structure made a hydrogen bond and thus contributed its tighter binding to the S1 site than the rhamnoside moiety of quercitrin (Jo et al., 2019). In another report, Jo et al. Jo et al. (2019a) distinguished several promising compounds that inhibit SARS-CoV-2 3CL^pro^: baicalin (IC_50_ = 34.71 μM), herbacetin (IC_50_ = 53.90 μM) and pectolinin (IC_50_ = 51.64 μM). Baicalin and pectolinin bind in a similar manner due to the presence of a glucuronide group. Interestingly, with SARS-CoV 3CL^pro^ the affinity of baicalin was low, the affinity was much stronger for SARS-CoV-2 3CLO^pro^, despite 96% sequence identity of the active sites of both enzymes. The unique binding method (including the π–π stacking between amino acid residue and the phenyl moiety) of this compound is quite promising in the future design of SARS-CoV-2 3CL^pro^ inhibitors (Jo et al., 2020). Other natural compounds with the ability to bind at the substrate binding pocket 3CLO^pro^ include ellagic acid (-8.4 kcal/mol), kaempferol (-7.8 kcal/mol) or quercetin (-7.5 kcal/mol) and rutin (-9.4 kcal/mol). As can be seen, rutin (quercetin glycoside and disaccharide rutinosis) has a lower, more favorable binding energy which is consistent with other observations in the model studies described. Moreover, the RMSD (root mean square deviation) and RMSF (root mean square fluctuation) values confirmed the formation of stable ligand-protein complexes with kaempferol, quercetin and rutin (Rehman et al., 2020). Interestingly, these compounds has a lower binding energy than the tested reference structures: chloroquine (-5.2 kcal/mol), hydroxychloroquine (-5.2 kcal/mol), remdesivir (-7.5 kcal/mol) and ivermectin (-9.3 kcal/mol) (Rehman et al., 2020).

It was found that the presence of rutinose disaccharide (α-L-rhamnopyranosyl-(1 → 6)-β-D-glucopyranose) at position no. 3 of C ring is required for binding to the N3 inhibitor 3CL^pro^ proteinase binding site. The presence of a rutinose residue at the 3-position of the C-ring and the absence of an O-methyl group in the B-ring of the flavonol structure may increase the bond stability (Owis et al., 2020). The stability of this bond is lower for isoramnnetine monoglucoside, which has an o-methyl moiety in the B ring, than for kaempferol monoglucoside (Figure 8) (Owis et al., 2020). Molecular docking analyzes also showed a low binding energy value for rutin (quercetin-3-O-rutinose, -9.2 kcal/mol) and other rutin glucuronide derivatives, nicotiflorin and sulfate derivatives (vs non covalent inhibitor x77–8.4 kcal/mol). For comparison, quercetin (-7.5 kcal/mol) and kaempferol (-7.2 kcal/mol) had less binding energy similar to that of 3-O-sulfates derivatives. Importantly, rutinose flavonoids undergo deglycosylation in the digestive system and are conjugated as aglycones with glucuronate and sulfate. These are the main chemical forms found in plasma (da Silva et al., 2020). Docking studies done against the active site of M^pro^ protein described by Cherrak et al. Cherrak et al. (2020) presented few flavonoid structures with highest affinity for the active site with binding energies: quercetin 3-rhamonoside (-9.7 kcal/mol), myricetin 3-rutinoside (-9.3 kcal/mol) and rutin (-9.2 kcal/mol). The most effective compounds had sugar moieties in their structure, and this feature seems to be important. Compounds substituted at the carbon no. 3 display higher activity than those substituted at the carbon no. Seven e.g., genistein (-7.5 kcal/mol). da Silva et al. da Silva et al. (2020) and Cherrak et al. Cherrak et al. (2020) claimed that the sugar moieties increase the bioavailability of the flavonoid.

Epigallocatechin gallate (EGCG) and gallocatechin gallate (GCG) showed inhibition of recombinant 3CL^pro^ with the value of IC_50_ 73 and 47 μM, respectively. The molecular modeling revealed that GCG competitively bound to the active site of the enzyme and it required galloyl moiety at the 3-OH position for this activity. The galloyl group from GCG was important for GCG binding to 3CL^pro^ active site pocket because it has four hydrogen bond interactions with amino acid residues (Nguyen et al., 2012). Among the phytochemicals tested *in silico* by Tahir ul Qamar et al. Tahir ul Qamar et al. (2020), 5,7,3′, 4′-tetrahydroxy-2'-(3,3-dimethylallyl) isoflavone, naturally occurring in *Psorothamnus arborescens*, exhibited the highest binding affinity (−29.57 kcal/mol) by forming strong hydrogen bonds with the catalytic dyad residues and receptor-binding residues. For comparison, the binding affinity of a conventional drug (nelfinavir) is -17.1 kcal/mol. Other compounds with relatively high affinity include myricitrin (−22.13 kcal/mol) and methyl rosmarinate (−20.62 kcal/mol). Interestingly, all three compounds formed stable ligand-protein complexes and internal SARS-CoV-2 hydrogen bonds. 3CL^pro^ remained main stable throughout the simulation, with no obvious fluctuations (Tahir ul Qamar et al., 2020). Many authors note that phenolic compounds constitute a rich reservoir of potential inhibitors of this protease. Many compounds analyzed *in silico* by Vijayakumar et al. Vijayakumar et al. (2020) showed a beneficial interaction with active or dimerisation sites or regulatory sites. As many as 22 relationships (among 22 interacting with M^pro^) were common sites, incl. kaempferol, quercetin, apigenin, cyanidin, naringenin, pelargonidin, fisetin etc. The best candidate turned out to be indole-chalcone with a binding affinity of −10.4 kcal/mol and, the flavonoid quercetin, −9.2 kcal/mol (Vijayakumar et al., 2020). Among the 14 flavonoids analyzed by Peterson (Peterson, 2020), the active site of 3CL^pro^ was the lowest binding energy of amentoflavone (average -8.8 kcal/mol) and diosmin and gallocatechin gallate (-8.4 kcal/mol), while baicalein (-7.3 kcal/mol) had the highest binding energy, as well as naringinin, hispidulin, homoplantaginin or fisetin. It was also suggested that combined therapy involving antiviral drug and flavonoid could give synergistic effect (Peterson, 2020). Another possible strategy is to inhibit the cation-selective channel formed by 3a protein, that may become expressed in the infected cell.

### Role of Phenolic Compounds in Interrupting the Viral Exocytosis and Virus Releasing

In studies by Schwarz et al. Schwartz et al. (2011) Schwarz et al. (2014), it was found that kaempferol and emodin inhibit the activity of the 3a channel, resulting in blockage of virus release from infected cells by exocytosis. Interestingly, quercetin does not have this property, even at high concentration. The presence of sugar residues in kaempferol derivatives enhanced the inhibitory activity (junglanin and apheline) (Schwartz et al., 2011; Schwarz et al., 2014).

After the virus enters the host cell, it replicates in a process involving RNA-dependent RNA polymerase (RdRp). *In silico* studies reveled that cyanidin showed H-bonding with Asp761 at the RNA binding site with a binding affinity of −7.7 kcal/mol, which can potentially disrupt replication (Vijayakumar et al., 2020). Ragavan Rameshkumar et al. Ragavan Rameshkumar et al. (2020) also described 36 flavonoid compounds, with binding energy value > -9 kcal/mol. Interestingly, the albireodelphin have binding energy -9.8 kcal/mol against RdRp. The best, five compounds were also identified according to ADMET (absorption, distribution, metabolism, excretion, and toxicity) and docking score as potent inhibitors against COVID-19 main protease, RdRp and spike protein (Ragavan Rameshkumar et al. 2020). Docking analyzes by Singh et al. Singh et al. (2020) indicated that some natural polyphenols form stable conformations with RdRp. For example, quercetagetin is docked at the SARS-CoV-2 RdRp active site with a favourable affinity for the binding pocket (Singh et al., 2020).

Described studies showed the important role of preliminary *in silico* research for effective compounds of plant origin against COVID-19. Due to the precise analysis of the flavonoid structure, it is possible to identify the functional groups responsible for specific and effective binding to viral macromolecules, inhibiting its penetration and replication.

In conclusion, there are reports in the literature showing that phenolic compounds are active against the SARS-CoV-2. However, most of these results were obtained from computational modelling and computational predictions. Anti-SARS-CoV-2 activity of phenolic compounds should be assessed scientifically and clinically (Goc et al., 2021).

## Antiviral Activity of Metal Complexes With Phenolic Compounds

In principle, there are no studies on the antiviral activity of metal complexes with phenolic compounds. There are literally only a handful of publications on this topic. The promising results of the research presented below should encourage scientists to develop this issue. This is especially important during the coronavirus pandemic, as these modern compounds may prove effective against SARS-CoV-2.

Langland et al. Langland et al. (2018) determined the effectiveness of the caffeic acid chelates with iron (III) against eight virus strains, including HSV-1 and vaccinia virus (VV). All tested viruses required attachment of glycoprotein B to heparan sulfate proteoglycan on the surface of the host cell in order to enter the cell. The conducted research showed that the antiviral properties of iron(III) and caffeic acid complexes were over 100 times stronger than the antiviral activity of caffeic acid alone. In addition, it was found that the molar ratio of iron(III) to caffeic acid had an effect on the antiviral properties. The higher it was, the stronger antiviral activity of the compound against the HSV-1 was reported. Studies have shown that even relatively small amounts of Fe(III) significantly increased the anti-HSV-1 properties of caffeic acid. Copper(II) and zinc ions also caused greater antiviral activity of caffeic acid. The authors concluded that metal chelation by catechol in caffeic acid enhanced the antiviral activity of caffeic acid. In addition, researchers supposed that caffeic acid chelates attacked and disrupted the process of binding the virus to receptors on the surface of the host cell. Such compounds in combination with existing drugs like acyclovir could be used to increase control over viral infections. However, further research on the mechanism of action of caffeic acid chelates against viruses is required (Langland et al., 2018).

Flavonoids are metal chelators as well. Literature reports confirmed that these phenolic compounds could bind metals such as iron or copper (Rodriguez et al., 2006).

Curcumin and its derivatives such as Gal (gallium)-curcumin and Cu (copper)-curcumin showed activity against HSV-1 (KOS strain) in Vero cell line. The IC_50_ values were: 33.0 μg/ml, 13.9 μg/ml and 23.1 μg/ml, respectively. The scientists emphasized that the mechanism of action of these compounds should be elucidated in future research (Zandi et al., 2010).

It has been proven that curcumin with copper (Cu(II)) ions was a good antiviral agent. This complex was active against viruses such as Coxsackie virus B4, vesicular stomatitis virus (VSV) and respiratory syncytial virus (RSV) (Chauhan et al., 2013).

**FIGURE 9:**
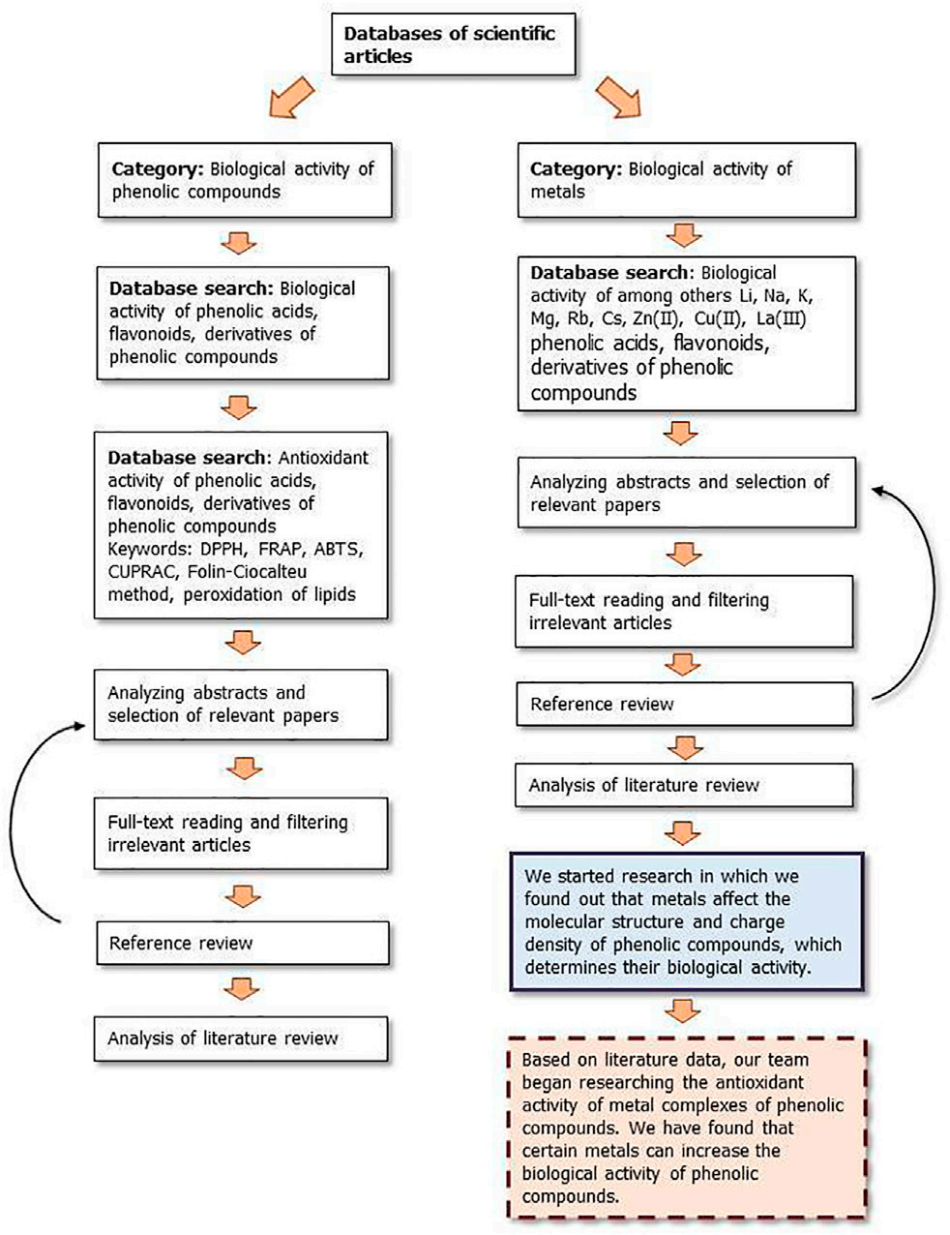
The flow diagram which show our strategy for literature survey (part I) based on Ivanov et al. (2019).

**FIGURE 10:**
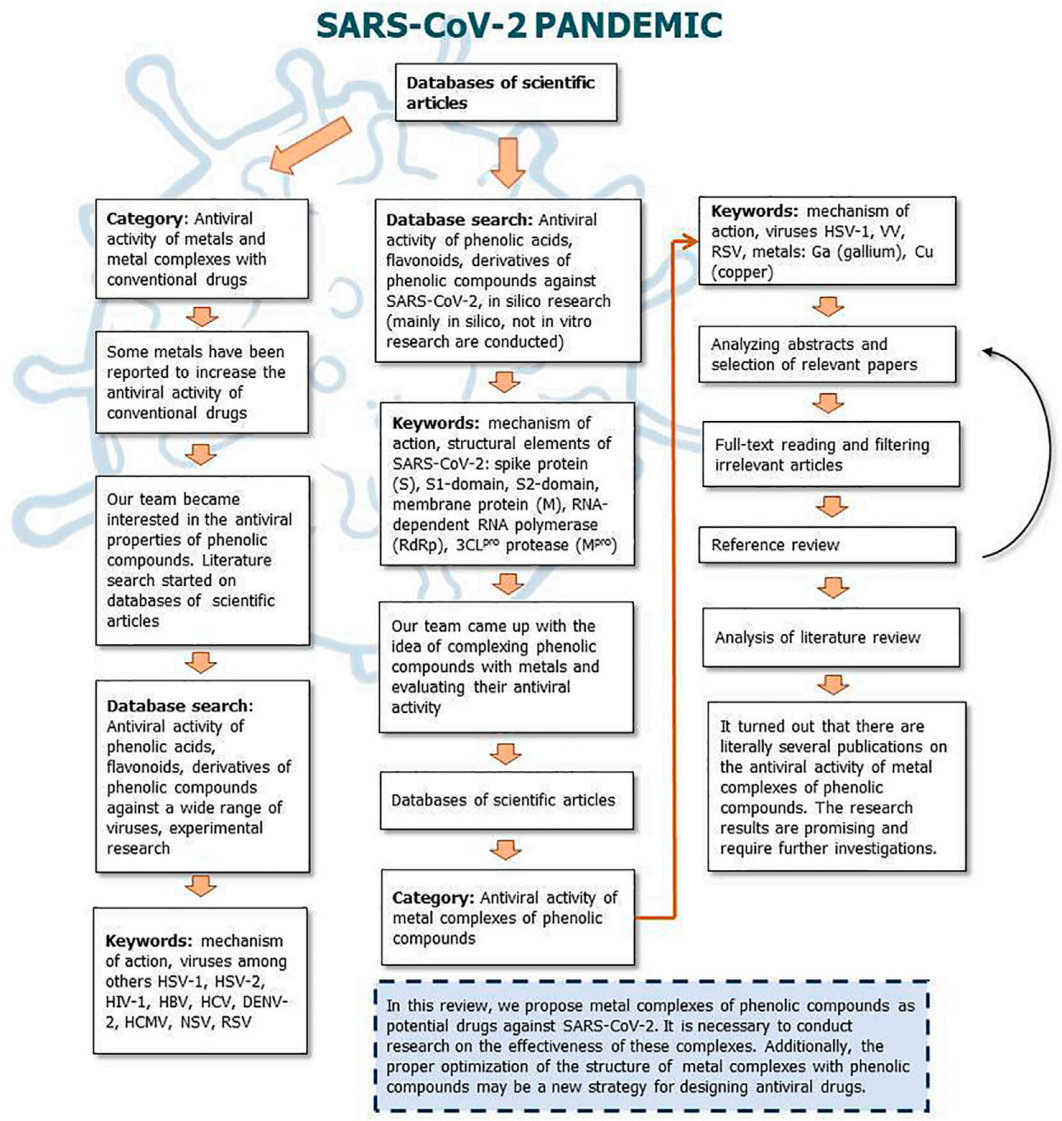
The flow diagram which show our strategy for literature survey (part II) based on Ivanov et al. (2019).

Figures 9, 10 show a flow diagram which show our strategy for literature survey for findings described in this publication.

## Perspectives Concerning Plant Phenolic Compounds and Their Derivatives

Phenolic compounds, due to various biological properties, such as antioxidant, antibacterial, antifungal, antiviral, anticancer, can find potential application in pharmacy and medicine (supplements, drugs) as well as food industry (food additives, preservatives) (Kalinowska et al., 2013; Samsonowicz et al., 2017). Polyphenols as well as plant extracts used for centuries in traditional medicine may become the basis for the development of drugs effective in the treatment and prevention of COVID-19 disease (Mehany et al., 2021). Currently, there is an increasing use of compounds of natural origin in biotechnology and medicine, as they have high-safety profile without causing major side effects. Their toxicity after ingestion still needs to be thoroughly investigated with metabolomics, proteomics and omics science-genomics (Mehany et al., 2021). Conducting research on the synthesis of new derivatives of phenolic compounds may contribute to obtaining new compounds with increased desirable biological properties (Kalinowska et al., 2016). The application of various analytical methods in conjunction with quantum-chemical calculations allows achieving information on the influence of metal ions on the antiviral properties of phenolic compounds (Samsonowicz et al., 2021). Thanks to this, it will be possible to select complexes with the strongest activity. In the next stage, biochemical- and cell-based assays must be carried out, followed by investigations *in vivo* and clinical trials, preceded by studies on the development of a method of delivering these compounds.

## Conclusion

The presented literature review shows that phenolic compounds such as chlorogenic, quinic, dicaffeoylquinic, gallic and ellagic acid, quercetin, catechin, epicatechin, myricetin, fisetin, chrysin, luteolin, apigenin, genistein, baicalein and naringenin exhibit antiviral properties. Importantly, plant phenolic compounds cause significantly less host cell damage than pharmaceuticals. Metal ions can enhance the antiviral activity of compounds of natural origin. In the design of antiviral substances based on metal complexes with plant phenolic compounds the following factors should be taken into account:

• the position and number of hydroxyl groups in the aromatic ring, the degree of the electronic charge delocalization, the length of the conjugated double bond system,
• the aromatic properties of the phenolic compounds, which are measured using the aromaticity indices and donor-acceptor properties described by the energy of LUMO (Lowest Unoccupied Molecular Orbital) and HOMO (Highest Occupied Molecular Orbital) orbitals,
• ionic radius, electronegativity, ionic potential and effective charge of metal ions.

The proper optimization of the structure of metal complexes with phenolic compounds may be a new strategy for designing antiviral drugs. The way polyphenols inhibit viral multiplication depends on the type of phenolic compound as well as the virus they act on. The compounds described in this study showed antiviral properties at various stages of the virus life cycle, i.e., attachment, penetration, replication, assembly and release, but they also contribute to the modifications in the biochemical processes of the host, which indirectly influence the virus life cycle (Si et al., 2007). Unfortunately, the mechanism of action of phenolic compounds on viruses is still not fully understood and requires further research. Despite of the positive impact of the phenolic compounds in the inhibition of viral infections, restrictions on the use of these compounds should also be raised. Many of the limitations of the use of phenolic compounds as antiviral drugs, such as low bioavailability, high extraction costs, and industrial refining remain of serious concern (Albuquerque et al., 2021). Other difficulties in phenolic compound applications may be due to their low stability and solubility. New technologies to improve the targeted release of phenolic compounds are still being developed (Esfanjani et al., 2018). Knowledge of the interactions between antiviral phenolic compounds or metal complexes with phytochemicals and their targets would enable the design of new inhibitors (Annunziata et al., 2020). Unfortunately, the mechanism of action of phenolic compounds on viruses is still not fully understood and requires further research. Knowledge of the interactions between antiviral phenolic compounds or metal complexes with phytochemicals and their targets would enable the design of new inhibitors (Annunziata et al., 2020).

## Footnotes

1 National Center for Biotechnology Information (2021). PubChem Compound Summary for CID 689043, Caffeic acid. Retrieved July 20, 2021 from https://pubchem.ncbi.nlm.nih.gov/compound/Caffeic-acid.

8 National Center for Biotechnology Information (2021). PubChem Compound Summary for CID 6508, Quinic acid. Retrieved July 20, 2021 from https://pubchem.ncbi.nlm.nih.gov/compound/Quinic-acid.

9 National Center for Biotechnology Information (2021). PubChem Compound Summary for CID 5280343, Quercetin. Retrieved July 20, 2021 from https://pubchem.ncbi.nlm.nih.gov/compound/Quercetin.

16 National Center for Biotechnology Information (2021). PubChem Compound Summary for CID 5281605, Baicalein. Retrieved July 20, 2021 from https://pubchem.ncbi.nlm.nih.gov/compound/Baicalein.

17 National Center for Biotechnology Information (2021). PubChem Compound Summary for CID 54758660, Fisetin(1-). Retrieved July 20, 2021 from https://pubchem.ncbi.nlm.nih.gov/compound/Fisetin_1.

25 National Center for Biotechnology Information (2021). PubChem Compound Summary for CID 439246, Naringenin. Retrieved July 20, 2021 from https://pubchem.ncbi.nlm.nih.gov/compound/Naringenin.

26 National Center for Biotechnology Information (2021). PubChem Compound Summary for CID 5280805, Rutin. Retrieved July 20, 2021 from https://pubchem.ncbi.nlm.nih.gov/compound/Rutin.

31 National Center for Biotechnology Information (2021). PubChem Compound Summary for CID 5352000, Myricetin 3-rhamnoside. Retrieved July 20, 2021 from https://pubchem.ncbi.nlm.nih.gov/compound/Myricetin-3-rhamnoside.

32 National Center for Biotechnology Information (2021). PubChem Compound Summary for CID 5487413, Myricetin 3-O-glucuronide. Retrieved July 20, 2021 from https://pubchem.ncbi.nlm.nih.gov/compound/Myricetin-3-O-glucuronide.

38 National Center for Biotechnology Information (2021). PubChem Compound Summary for CID 73399, Pinoresinol. Retrieved July 20, 2021 from https://pubchem.ncbi.nlm.nih.gov/compound/Pinoresinol.

39 National Center for Biotechnology Information (2021). PubChem Compound Summary for CID 5281647, Mangiferin. Retrieved July 20, 2021 from https://pubchem.ncbi.nlm.nih.gov/compound/Mangiferin.

43 National Center for Biotechnology Information (2021). PubChem Compound Summary for CID 122738, Procyanidin B2. Retrieved July 20, 2021 from https://pubchem.ncbi.nlm.nih.gov/compound/Procyanidin-B2.

44 Sigma-Aldrich. Dihydrokaempferol. Retrieved July 20, 2021. https://www.sigmaaldrich.com/PL/pl/product/sial/92204

45 Sigma-Aldrich. Phloridzin dihydrate. Retrieved July 20, 2021. https://www.sigmaaldrich.com/PL/pl/product/aldrich/274313

## Glossary

5-HNQ: 5-hydroxynaphthoquinone
ACE2: angiotensin-converting enzyme 2
Ad 2: human adenovirus type 2
ADMET: absorption, distribution, metabolism, excretion, and toxicity
ADV: adenovirus
BDE: bond dissociation energy
CHA: chlorogenic acid
COVID-19: Coronavirus disease 2019
CPE: cytopathic effect
CVB3: Coxsackie virus B3
CC50: 50% cytotoxic concentration
DENV-2: Dengue virus type 2
DHNQ: 5,8-dihydroxy-1,4-naphthoquinone
EC50: 50% effective concentration
ED50: 50% effective dose
EGCG: epigallocatechin gallate
FMDV: foot-and-mouth disease virus
GCG: epigallocatechin gallate
HBV: hepatitis B virus
HCMV: human cytomegalovirus
HCV: hepatitis C virus
HIV: human immunodeficiency virus
HO-1: heme oxygenase
HSV: herpes simplex virus
IC50: 50% inhibitory concentration
IN: integrase
IP: ionization potential
LD50: 50% lethal dose
NA: neuraminidase
NGC strain: New Guinea C strain
NP: nucleoprotein
NQ: naphthoquinone
NSV: Sindbis virus neurovirulent strain
pp: pseudoparticles
QSAR: quantitative structure–activity relationship
RdRp: RNA-dependent RNA polymerase
RMSD: root mean square deviation
RMSF: root mean square fluctuation
RSV: respiratory syncytial virus
RT: reverse transcriptase
SARS-CoV-2: Severe acute respiratory syndrome coronavirus 2
SI: selectivity index
SIV: Simian immunodeficiency virus
TI: therapeutic index
VSV: vesicular stomatitis virus
VV: vaccinia virus
