# Supplementary material for: Drug Design Strategies for the Treatment of Viral Disease. Plant Phenolic Compounds and Their Derivatives
Source: Front Pharmacol. 2021 Jul 30;12:709104. doi: 10.3389/fphar.2021.709104 (PMC8363300; doi:10.3389/fphar.2021.709104)
Supplement: Supplementary file 1 [file DataSheet1.PDF]

Supplementary Material

1 Supplementary Tables

Supplementary Table 1. Antiviral activity of phenolic compounds and its derivatives (developed on the basis of cell-based assays).

**Phenolic compound/Plant extract**	Virus	**Cell line/*In vivo* model**	**Activity of compound**	**Mechanism of action/Biological action**	**References**
**Caffeic acid**[1] (3,4-dihydroxycinnamic acid)	HSV-1 (F strain)	HEp-2	C=4 mM (reduction of virus yield about 1000 times)	Probable inhibition of proteins relevant for viral-host interactions and for replication	(Ikeda et al., 2011)
		MDCK	EC_{50}=0.4-0.8 µg/ml	Not described in (Özçelik et al., 2011)	(Özçelik et al., 2011)
	Parainfluenza virus type-3	Vero	EC_{50}>0.8 µg/ml	Not described in (Özçelik et al., 2011)	(Özçelik et al., 2011)
**Quinic acid**[1]	HSV-1	MDCK	EC_{50}=0.05-0.8 µg/ml	Not described in (Özçelik et al., 2011)	(Özçelik et al., 2011)
	Parainfluenza virus type-3	Vero	EC_{50}=0.4-1.6 µg/ml	Not described in (Özçelik et al., 2011)	(Özçelik et al., 2011)
**Chlorogenic acid**[1] (5-caffeoylquinic acid)	Influenza virus (subtype H1N1)	MDCK	EC_{50}=44.87±1.12 µM	Inhibition of neuraminidase (NA), reduction of the NP viral protein and its nuclear retention	(Ding et al., 2017)
		A lethal murine	100 mg/kg/d, survival rate		

		infection model	60%		
	Influenza virus (subtype H3N2)	MDCK	EC_{50}=62.33±1.22 μM		
		A lethal murine infection model	100 mg/kg/d, survival rate 50%		
	HSV-1	MDCK	EC_{50}=0.4-0.8 μg/ml	Not described in (Özçelik et al., 2011)	(Özçelik et al., 2011)
	Parainfluenza virus type-3	Vero	EC_{50}=0.4-1.6 μg/ml	Not described in (Özçelik et al., 2011)	(Özçelik et al., 2011)
**Chebulagic acid**[1]	HSV-2	Vero	IC_{50}=1.41±0.51 μg/ml	Prevention the attachment and penetration of virus into cells	(Kesharwani et al., 2017)
**Chebulinic acid**[1]	HSV-2	Vero	IC_{50}=0.06±0.002 μg/ml	Prevention the attachment and penetration of virus into cells	(Kesharwani et al., 2017)
**Gallic acid**[1]	HSV-1	MDCK	EC_{50}=0.05-0.8 μg/ml	Not described in (Özçelik et al., 2011)	(Özçelik et al., 2011)
	Parainfluenza virus type-3	Vero	EC_{50}=0.05-0.8 μg/ml	Not described in (Özçelik et al., 2011)	(Özçelik et al., 2011)
	HIV-1	TZM-b1	IC_{50}=0.36 μg/ml	Inhibition of RT (reverse transcriptase)	(Nutan et al., 2012)
		CEM-GFP	IC_{50}=0.19 μg/ml	Inhibition of RT	(Nutan et al., 2012)

	HIV-1 (III_B strain)	C8166	EC_{50}=6.09 μg/ml	Inhibition of p24 antigen production; inhibition of HIV-1 entry (targeting gp41); inhibition of HIV-1 (III_B strain) replication (targeting viral proteins mediating late stages of viral replication);	(Rashed et al., 2012)
**Ellagic acid**[1]	HIV-1	TZM-b1	IC_{50}=18.8 μg/ml	Inhibition of HIV-1 protease	(Nutan et al., 2012)
		CEM-GFP	IC_{50}=73 μg/ml	Inhibition of HIV-1 protease	(Nutan et al., 2012)
	HIV-1 (III_B strain)	C8166	EC_{50}=12.32 μg/ml	Inhibition of HIV-1 (III_B strain) replication	(Rashed et al., 2012)
**3,4- O-dicaffeoylquinic acid**[1]	HBV	HepG2.2.15 cells	C=50-100 μg/ml	Inhibition the expression of HBsAg and HBeAg, reduction the amount of HBV cccDNA, upregulation of heme oxygenase 1 (HO-1)	(Wu et al., 2012)
**Quercetin**[2]	HSV-1	MDCK	CPE inhibitory concentration 0.1-0.2 μg/ml	Not described in (Özçelik et al., 2011)	(Özçelik et al., 2011)
		Vero	EC_{50}=5 μM	Not described in (Lyu et al., 2005)	(Lyu et al., 2005)
	Parainfluenza virus type-3	Vero	No activity	-	(Özçelik et al., 2011)
	DENV-2 (New Guinea C strain)	Vero	C=50 μg/ml (14±1.5% reduction of DENV-2 foci)	Prevention of viral infection (prophylactic activity)	(Zandi et al., 2011)
			IC_{50} (50% inhibitory	Anti-virus activity in the post-adsorption stage;	

			concentration)=35.7 µg/ml	blocking DENV-2 replication	
			IC_{50}=28.9 µg/ml	Antiviral activity in the period from 5 days before infection to 4 days after infection; blocking DENV-2 replication	
	HIV-1 (III_B strain)	C8166	EC_{50}=42.55 µg/ml	Inhibition of HIV-1 (III_B strain) replication	(Rashed et al., 2012)
**Myricetin**[2]	HSV-1	Vero	EC_{50}=5 µM	Not described in (Lyu et al., 2005)	(Lyu et al., 2005)
	HIV-1 (III_B strain)	C8166	EC_{50}=28.37 µg/ml	Inhibition of HIV-1 (III_B strain) replication	(Rashed et al., 2012)
**Fisetin**[2]	DENV-2 (New Guinea C strain)	Vero	IC_{50}=55 µg/ml (after virus adsorption); IC_{50}=43.12 µg/ml (5 h before virus infection and continuously up to 4 days post-infection)	Reduction the level of DENV-2 RNA; no direct virucidal or prophylactic activity	(Keivan et al., 2014)
**Kaempferol**[2]	HSV-1	Vero	EC_{50}=15 µM	Not described in (Lyu et al., 2005)	(Lyu et al., 2005)
**Catechin**[3]	HSV-1	Vero	EC_{50}=4 µM	Not described in (Lyu et al., 2005)	(Lyu et al., 2005)
**Epicatechin**[3]	HSV-1	Vero	EC_{50}=2.5 µM	Not described in (Lyu et al., 2005)	(Lyu et al., 2005)
**Daidzein**[4]	DENV-2 (New Guinea C strain)	Vero	IC_{50}=142.6 µg/ml	Very weak anti-virus activity in the post-adsorption stage; blocking DENV-2 replication	(Zandi et al., 2011)
			C=50 µg/ml (about 25% reduction in viral RNA copy	Very weak anti-virus activity in the period from 5 days before infection to 4 days after infection;	

			number)	blocking DENV-2 replication	
	Influenza virus A/Jinan/15/90 (H3N2) strain	MDCK	IC_{50}>787 µM	Inhibition of viral NA activity	(Liu et al., 2008)
**Apigenin**[4]	HSV-1	MDCK	EC_{50}=0.4-1.6 µg/ml	Not described in (Özçelik et al., 2011)	(Özçelik et al., 2011)
	Parainfluenza virus type-3	Vero	EC_{50}>0.2 µg/ml	Not described in (Özçelik et al., 2011)	(Özçelik et al., 2011)
	Influenza virus A/Jinan/15/90 (H3N2) strain	MDCK	IC_{50}=4.74 µM	Inhibition of viral NA activity	(Liu et al., 2008)
**Luteolin**[4]	Influenza virus A/Jinan/15/90 (H3N2) strain	MDCK	IC_{50}=6.82 µM	Inhibition of viral NA activity	(Liu et al., 2008)
**Baicalein**[4]	Human cytomegalovirus (HCMV) (Towne strain)	Human embryonic lung fibroblasts (HEL 299)	IC_{50}=0.4-1.2 µM	Reduction the level of HCMV immediate-early, early and late proteins and viral DNA synthesis, blocking epidermal growth factor receptor tyrosine kinase activity and HCMV nuclear translocation; primary mechanism of action - inhibition of viral entry into the cell	(Evers et al., 2005)
**Chrysin**[4]	HSV-1	Vero	EC_{50}=2.5 µM	Not described in (Lyu et al., 2005)	(Lyu et al., 2005)
**Genistein**[5]	HSV-1	MDCK	EC_{50}=0.4-0.8 µg/ml	Not described in (Özçelik et al., 2011)	(Özçelik et al., 2011)
	Parainfluenza virus	Vero	EC_{50}=0.2-0.4 µg/ml	Not described in (Özçelik et al., 2011)	(Özçelik et

	type-3				al., 2011)
	HCMV (Towne strain)	HEL 299	IC_{50}=3.2-38 µM	Reduction the level of HCMV early and late proteins and viral DNA synthesis; primary mechanism of action - blocking HCMV immediate-early protein functioning	(Evers et al., 2005)
	HSV-1	Vero	EC_{50}=5 µM	Not described in (Lyu et al., 2005)	(Lyu et al., 2005)
**Naringenin**[6]	Sindbis virus neurovirulent strain (NSV)	BHK-21 clone 15 cells	ID_{50}=14.5-14.9 µg/ml	Inhibition of Sindbis virus neurovirulent strain (NSV) replication	(Paredes et al., 2003)
**Hesperetin**[6]	DENV-2 (New Guinea C strain)	Vero	No activity when administered after adsorption of virus to cells	-	(Zandi et al., 2011)
			C=50 µg/ml (about 2% reduction in viral RNA copy number)	No significant antiviral activity in the period from 5 days before infection to 4 days after infection	
	NSV	BHK-21 clone 15 cells	ID_{50}=15.9-20.5 µg/ml	Inhibition of Sindbis virus neurovirulent strain (NSV) replication	(Paredes et al., 2003)
**Naringin**[7]	Parainfluenza virus type-3	Vero	EC_{50} >0.2 µg/ml	Not described in (Özçelik et al., 2011)	(Özçelik et al., 2011)
	DENV-2 (New Guinea C strain)	Vero	IC_{50}=168.2 µg/ml; C=50 µg/ml (about 25.8±0.76% reduction in DENV-2 RNA production)	Blocking the step of viral adsorption into cells	(Zandi et al., 2011)

			No activity when administered after adsorption of virus to cells	-	
			C=50 µg/ml (about 5% reduction in viral RNA copy number)	No significant antiviral activity in the period from 5 days before infection to 4 days after infection	
**Baicalin**[7]	HSV-1	Vero	EC_{50}=5 µM	Not described in (Lyu et al., 2005)	(Lyu et al., 2005)
**Silibinin**[7]	HSV-1	MDCK	EC_{50}=0.1-0.4 µg/ml	Not described in (Özçelik et al., 2011)	(Özçelik et al., 2011)
	Parainfluenza virus type-3	Vero	EC_{50}>0.4 µg/ml	Not described in (Özçelik et al., 2011)	(Özçelik et al., 2011)
**Silymarin**[7]	HSV-1	MDCK	EC_{50}=0.8-1.6 µg/ml	Not described in (Özçelik et al., 2011)	(Özçelik et al., 2011)
	Parainfluenza virus type-3	Vero	No activity	-	(Jo et al., 2019)
	Hepatitis C virus (HCV)	Huh-7.5	C=80 µM (about 80% inhibition of entry of pseudotyped viruses)	Inhibition of virus entry into the cell (inhibition the entry of viral pseudoparticles, HCVpp)	(Wagoner et al., 2010)
		Huh-7.5.1	C=40 µM (significant reduction in RNA production at 48 and 72 hours after treatment)	Inhibition of RNA production	

		Huh-7.5.1	C=80 µM (reduction of MTP activity by about 20% in 14 day chronically infected cells)	Inhibition of microsomal triglyceride transfer protein (MTP) activity	
		Huh-7.5.1, HepG2	C=80 µM (about 0.9 and 0.4 fold change in apoB, respectively)	Inhibition of secretion of apolipoprotein B (apoB) from HCV-infected cells	
		Huh-7.5.1	C=40 µM (five and two times reduction in infectious virus yields at 48 and 72 hours post infection)	Inhibition of infectious virion production	
		Huh-7.5	C=80 µM (inhibition of cell-to-cell spread of virus by about 35%)	Inhibition of cell-to-cell spread of virus	
**quercetin-3-O-β-D-xylopyranosyl (1 → 2) α-L-rhamnopyranoside**[7]	HSV-1	Vero	EC_{50}=5.8 µg/ml	Not described in (Ürményi et al., 2016)	(Ürményi et al., 2016)
	HSV-2	Vero	EC_{50}=36.2 µg/ml	Not described in (Ürményi et al., 2016)	(Ürményi et al., 2016)
**kaempferol-3-O-β-D-xylopyranosyl (1 → 2) α-L-rhamnopyranoside**[7]	HSV-1	Vero	EC_{50}=7.4 µg/ml	Not described in (Ürményi et al., 2016)	(Ürményi et al., 2016)
	HSV-2	Vero	EC_{50}=9.0 µg/ml	Not described in (Ürményi et al., 2016)	(Ürményi et al., 2016)
**Kaempferol-3-O-(6”-O-E-p-coumaroyl)-β-D-glucopyranoside**[7]	Respiratory syncytial virus (RSV) (Long strain)	HEp-2	IC_{50}=6.3±0.2 µM	Not described in (Zhang et al., 2015)	(Zhang et al., 2015)

**Methyl gallate**[7]	HIV-1 (III_B strain)	C8166	EC_{50}=20.53 µg/ml	Inhibition of HIV-1 (III_B strain) replication	(Rashed et al., 2012)
**Myricetin-3-O-α-rhamnoside**[7]	HIV-1 (III_B strain)	C8166	EC_{50}=14.15 µg/ml	Inhibition of HIV-1 (III_B strain) replication	(Rashed et al., 2012)
	HIV-1	MT4 cells	IC_{50}=10.6 µM	Inhibition of RT activity	(Ortega et al., 2017)
**Myricetin 3-(6-rhamnosylgalactoside)**[7]	HIV-1	MT4 cells	IC_{50}=13.8 µM	Inhibition of RT activity	(Ortega et al., 2017)
**Myricetin-3-O-α-glucuronide**[7]	HIV-1 (III_B strain)	C8166	EC_{50}=16.51 µg/ml	Inhibition of HIV-1 (III_B strain) replication	(Rashed et al., 2012)
**Sulfuretin**[7]	Influenza virus A/Jinan/15/90 (H3N2) strain	MDCK	IC_{50}>9.15 µM	Inhibition of viral NA activity	(Liu et al., 2008)
**Acacetin 7-O-[4'''-O-acetyl-β-D-apiofuransyl-(1→3)]-β-D-xylopyranoside**[7]	Respiratory syncytial virus (RSV) (A2 strain)	HEp-2	IC_{50}=81.7 µM	Not described in (Zhang et al., 2014)	(Zhang et al., 2014)
**Acacetin-7-O-[6'''-O-acetyl-β-D-galactopyranosyl-(1→2)]-β-D-glucopyranoside**[7]	HSV-1	Vero	IC_{50}=38.5 µM	Not described in (Zhang et al., 2014)	(Zhang et al., 2014)
**Mangiferin**[7]	RSV (Long strain)	HEp-2	IC_{50}=40.0±0.7 µM	Not described in (Zhang et al., 2015)	(Zhang et al., 2015)
**Methanolic extract of**	HSV-1	Vero	EC_{50}=710.9±1.6 µg/ml	Not described in (Suárez et al., 2010)	(Suárez et

**apple pomace**[8]					al., 2010)
	HSV-2	Vero	EC_{50}=629.6±50.7 µg/ml	Not described in (Suárez et al., 2010)	(Suárez et al., 2010)
**Acetonic extract of apple pomace**[8]	HSV-1	Vero	EC_{50}=576.7±17.2 µg/ml	Not described in (Suárez et al., 2010)	(Suárez et al., 2010)
	HSV-2	Vero	EC_{50}=450.7±40.8 µg/ml	Not described in (Suárez et al., 2010)	(Suárez et al., 2010)

[1]Phenolic acid; [2]Flavonoid (flavon-3-ol); [3]Flavonoid (flavan-3-ol); [4]Flavonoid (flavone); [5]Flavonoid (isoflavone); [6]Flavonoid (flavanone); [7]Derivative of phenolic compound; [8]Plant extract.

Supplementary Table 2. Antiviral activity of phenolic compounds (developed on the basis of biochemical-based assays).

Phenolic compound	Virus	Activity of compound	Mechanism of action/Biological action	References
**Apigenin**[1]	Influenza virus A/Jinan/15/90 (H3N2) strain	IC_{50}=28.9±0.7 µM	Inhibition of viral neuraminidase (NA) activity	(Liu et al., 2008)
**Luteolin**[1]	Influenza virus A/Jinan/15/90 (H3N2) strain	IC_{50}=32.6±0.1 µM	Inhibition of viral NA activity	(Liu et al., 2008)
**Daidzein**[1]	Influenza virus A/Jinan/15/90 (H3N2) strain	IC_{50}=26.6±0.3 µM	Inhibition of viral NA activity	(Liu et al., 2008)

**Sulfuretin**[2]	Influenza virus A/Jinan/15/90 (H3N2) strain	IC_{50}=27.7±0.8 μM	Inhibition of viral NA activity	(Liu et al., 2008)
**Silymarin**[2]	Hepatitis C virus (HCV)	IC_{50}=5 μM	Inhibition the fusion of viral pseudoparticles (HCVpp) with liposomes	(Wagoner et al., 2010)
		IC_{50}=300 μM	Inhibition of genotype 2a NS5B RNA-dependent RNA polymerase (RdRp) activity	

[1]Flavonoid (flavone); [2]Derivative of phenolic compound.

Abbreviations: apoB, apolipoprotein B; BHK, baby hamster kidney; cccDNA, closed covalent circular DNA; CPE, cytopathic effect; DENV-2, Dengue virus type 2; EC_{50}, 50% effective concentration; HBeAg, hepatitis B e antigen; HBsAg, hepatitis B surface antigen; HBV, hepatitis B virus; HCMV, human cytomegalovirus; HCV, hepatitis C virus; HEL 299, human embryonic lung fibroblasts; HEp-2, human epidermoid carcinoma #2 or human epithelioma-2; HIV, human immunodeficiency virus; HSV, herpes simplex virus; IC_{50}, 50% inhibitory concentration; ID_{50}, infectious Dose; MDCK, Madin-Darby canine kidney; MTP, microsomal triglyceride transfer protein; NA, neuraminidase; NP, nucleoprotein; NSV, Sindbis virus neurovirulent strain; RSV, respiratory syncytial virus; RT, reverse transcriptase;
